# Endoplasmic reticulum stress-induced CRELD2 promotes APMAP-mediated activation of TGF-β/SMAD and NF-κB pathways in esophageal squamous cell carcinoma

**DOI:** 10.3389/fimmu.2025.1616201

**Published:** 2025-07-31

**Authors:** Fangyu Su, Xia Yang, Zhaoyang Yan, Junhong Wu, Xiaoxu Li, Tongxin Xu, Huanchen Xu, Xinhao Wang, Zhaokun Hu, Juntao Lu, Wei Guo

**Affiliations:** ^1^ Laboratory of Pathology, Hebei Cancer Institute, the Fourth Hospital of Hebei Medical University, Shijiazhuang, Hebei, China; ^2^ Department of Radiation Oncology, Luoyang Branch of Dongzhimen Hospital Affiliated to Beijing University of Chinese Medicine, Luoyang Hospital of Traditional Chinese Medicine, Luoyang, Henan, China; ^3^ Department of Thoracic Surgery, the Fourth Hospital of Hebei Medical University, Shijiazhuang, Hebei, China; ^4^ Department of Radiation Oncology, the Fourth Hospital of Hebei Medical University, Shijiazhuang, Hebei, China; ^5^ Department of Computed Tomography and Magnetic Resonance Imaging (CT & MRI), the Fourth Hospital of Hebei Medical University, Shijiazhuang, Hebei, China

**Keywords:** esophageal squamous cell carcinoma, endoplasmic reticulum stress, CRELD2, APMAP, TGF-β/SMAD pathway, NF-κB pathway

## Abstract

**Background:**

Tumor cells experience endoplasmic reticulum (ER) stress due to oncogene activation and stressors in the tumor microenvironment, such as hypoxia and acidosis. ER stress plays a crucial role in carcinogenesis. However, its oncogenic mechanism in esophageal squamous cell carcinoma (ESCC) remains poorly understood.

**Methods:**

The transcriptional regulation of CRELD2 by ATF4 was investigated using a dual-luciferase reporter assay. Cellular proliferation, migration, and invasion capacities of ESCC cells were systematically evaluated through assays of MTS, colony formation, wound healing, transwell invasion, and flow cytometry analysis. To elucidate the molecular mechanisms underlying CRELD2 regulation, a series of experimental approaches including immunofluorescence, qRT-PCR, Western blotting, and co-immunoprecipitation assays were performed.

**Results:**

CRELD2 was identified as a significantly differentially expressed gene in ER-stressed ESCC cells, with its induction was mediated through the PERK-ATF4 pathway. CRELD2 exhibited oncogenic properties by enhancing ESCC cells proliferation, migration, and invasion, while also serving as a critical mediator of ER stress-regulated malignant behaviors. CRELD2 facilitated physical interaction with APMAP and promoted its cell membrane localization under ER stress. Notably, knockdown of APMAP significantly attenuated malignant phenotypes, mirroring the effects of CRELD2 depletion. Further investigations uncovered that APMAP activated TGF-β/SMAD pathway by binding to TAK1 in competition with transforming growth factor beta receptor I (TGFBR1). Concurrently, APMAP orchestrated TAK1/NF-κB signaling by enhancing TAK1 phosphorylation via facilitating the assembly of TAK1-TAB1-TAB2 ternary complexes.

**Conclusions:**

CRELD2, induced by the PERK-ATF4 pathway under ER stress, promotes localization of APMAP on the cell membrane, which subsequently triggers activation of TGF-β/SMAD and NF-κB signaling pathway, ultimately driving epithelial-mesenchymal transition and malignant progression of ESCC cells, and CRELD2 may serve as a promising therapeutic target for ESCC.

## 1 Introduction

Esophageal carcinoma is one of the most prevalent malignancies worldwide and the seventh leading cause of cancer-related deaths (1). It comprises two main subtypes: esophageal squamous cell carcinoma (ESCC) and esophageal adenocarcinoma (EAC), which differ in their epidemiology and pathophysiology. ESCC accounts for the majority of all esophageal cancers (2, 3). Despite advancements in early diagnosis and treatment, the 5-year survival rate of ESCC patients remains unsatisfactory (< 30%) (3). Thus, identifying reliable biomarkers is crucial for early diagnosis, treatment, and metastasis prevention of ESCC patients.

The endoplasmic reticulum (ER) serves as the primary site for protein synthesis, folding, and post-translational processing. During tumor initiation and progression, cancer cells experience diverse intra- and extracellular stressors, including intracellular oncogene activation, tumor suppressor loss, and microenvironmental challenges (4), and these stresses may disrupt proteostasis, lead to the accumulation of unfolded or misfolded proteins within the ER lumen and subsequently trigger ER stress (5). Recent studies have shown that the unfolded protein response (UPR) is activated in many human cancers and plays important roles in restoring cellular homeostasis (6). UPR effectors, including ERN1 (IRE1α), ATF6, and PERK (EIF2AK3), dissociate from HSPA5 (GRP78) and activate their respective canonical targets (XBP1s, ATF6α, and ATF4); subsequently trigger transcriptional reprogramming that promotes the survival of cancer cells under ER stress (7). However, prolonged or uncontrolled ER stress may induce apoptosis and inhibits tumor growth (5). Given the dual role of ER stress in cancer, it is crucial to understand its key regulatory mechanisms in tumor progression. Although ER stress has been implicated in various cancers, its oncogenic mechanisms in ESCC occurrence and progression remain incompletely understood and need to be further investigated.

Numerous ER stress-responsive genes regulated by the three branches of the UPR have been identified (8, 9). To explore the role of ER stress in ESCC, we performed RNA sequencing in an ER stress model of ESCC cells and focused on cysteine-rich with EGF-like domains 2 (CRELD2) among the differentially expressed genes, owing to its significant differential expression and functional relevance. CRELD2, primarily localized in the ER and Golgi apparatus, functions as both an ER-resident and secreted factor involved in protein folding and translocation (10). Recent studies have implicated CRELD2 as a potential oncogene in multiple cancers (11–14). For instance, abnormal glycosylation in tumor tissues facilitates CRELD2 secretion, promoting the progression of colorectal cancer (14). CRELD2 also drives tumor progression and is necessary for ROCK-induced education of cancer-associated fibroblasts to a tumor-promoting phenotype in breast cancer and cutaneous squamous cell carcinoma (12). Despite these findings, the role of CRELD2 in ESCC progression and its regulatory mechanisms under ER stress remain unclear.

In this study, we investigated the UPR branch responsible for CRELD2 induction in ESCC cells under ER stress and explored the biological functions and molecular mechanisms of CRELD2 in ESCC malignant progression.

## 2 Materials and methods

### 2.1 Cell culture and treatment

The human ESCC cell lines (TE1, KYSE150, and KYSE170) and the human normal esophageal epithelial cell line (HEEC) were obtained from the China Center for Type Culture Collection (CCTCC, Wuhan, China). The ESCC cell lines were maintained in RPMI 1640 medium (Invitrogen, Carlsbad, CA, USA) supplemented with 10% fetal bovine serum (FBS; Invitrogen), while HEEC cells were cultured according to the manufacturer’s instructions. These cells were detected and identified as free mycoplasma and bacteria infection during the past three months. The aforementioned cells were cultured in a humidified incubator at 37°C under 5% CO_2_ atmosphere. For thapsigargin (Tg) (#586005, MedChemExpress, Monmouth Junction, NJ, USA) or GSK2606414 (HY-18072, MedChemExpress) treatment, cells at 70% confluence were incubated with the specified concentrations of Tg or GSK2606414 for indicated durations, using DMSO (0.1%) as vehicle control.

### 2.2 RNA isolation, cDNA synthesis, and quantitative real time-PCR

Total RNA was extracted using TRIzol reagent (Solarbio, Beijing, China), and cDNA was synthesized with the MightyScript First Strand cDNA Synthesis Master Mix (Sangon Biotech, Shanghai, China), following the manufacturer’s protocol. Real-time PCR analyses were conducted with primer sets as described in **Supplementary Table S1**, employing SYBR Green real-time fluorescent quantitative PCR premix (Sangon Biotech) on the StepOne plus Real-Time PCR System (Applied Biosystems, Waltham, Massachusetts, USA). Relative gene expression levels were analyzed using the 2^–ΔΔCT^ method, with GAPDH serving as an internal reference for normalizing gene expression.

### 2.3 RNA-sequencing analysis

TE1 cells, treated with Tg or DMSO, were subjected to RNA-Sequencing analysis using the Illumina platform at Sangon Biotech. Differentially expressed genes (DEGs) were identified using DEseq R, applying the following criteria: |log2 (fold change)| > 1.

### 2.4 Transient transfection

Lipofectamine 2000 (Invitrogen) was used to perform the transient transfection. The siRNAs for PERK, IRE1α, ATF6, CREDL2, APMAP and siControl were purchased from GenePharma (Shanghai, China). Following transfection with 50 nM siRNA, cells were analyzed at 48 h post-transfection. For Tg treatment, transfected cells were re-plated and allowed to attach before being treated with either 100 nM Tg (+) or DMSO (−) for 12 h prior to analysis. The sequences of these siRNAs were listed in **Supplementary Table S2**. The pcDNA3.1-CRELD2 vector was acquired from Sangon Biotech. The human full-length cDNA fragments of ATF4 and APMAP were amplified and subsequently inserted into pcDNA3.1 vector (Invitrogen) respectively. The primers used were described in **Supplementary Table S3**. The transfection efficiency was assessed by qRT-PCR.

### 2.5 Dual-luciferase reporter assays

The CRELD2 promoter was cloned into the pGL3-Basic vector (Promega, Madison, WI, USA). KYSE150 cells were plated in 6-well plates and the pGL3-CRELD2 promoter luciferase plasmid was co-transfected with pcDNA3.1-ATF4, pcDNA3.1 empty plasmid and SV40 Renilla luciferase plasmid. After 48 h, luciferase activity was measured sequentially in each well using the Dual-Luciferase Reporter Assay System (Promega) and normalized to Renilla luciferase activity (control). The primers for various CRELD2 promoter fragments were listed in **Supplementary Table S4**.

### 2.6 Co-immunoprecipitation and Western blotting

For co-immunoprecipitation (CO-IP), the treated cells were lysed using NP-40 lysis buffer (Solarbio) supplemented with phenylmethanesulfonyl fluoride (PMSF) (Solarbio). Cellular proteins were clarified by centrifugation at 12,000 rpm. Protein-protein interactions were cross-linked by incubating the lysate supernatants with the indicated antibodies for 3 h at 4°C with constant rotation. Normal rabbit IgG (Abbkine, Wuhan, China) without antigenicity was used as a negative control. The complexes were then incubated with precleared protein A/G agarose beads (MedChemExpress) overnight at 4°C under gentle rotation. The immunoprecipitation complexes were boiled with sample loading buffer after three washes with NP-40 buffer. Proteins were separated by SDS-PAGE, transferred to polyvinylidene fluoride membranes (Millipore, Sigma, Burlington, MA, USA), and blocked with 5% skim milk in Tris-buffered saline (TBS). Immunodetection was performed with the specific primary antibodies for overnight incubation at 4 °C, followed by incubation with HRP-conjugated secondary antibodies for 1 h at room temperature. The protein bands were visualized via enhanced chemiluminescence (Zenbio).

For analysis of plasma membrane protein expression, plasma membrane was isolated using the MinuteTM Plasma Membrane Protein Isolation and Cell Fractionation Kit (SM-005 Invent Biotechnologies, Plymouth, MN, USA) following the manufacturer’s protocol. Protein expression levels were then determined by Western blot analysis.

The relative protein expression levels were quantified by densitometric analysis of Western blot bands using Image J software and normalized to β-actin or ATP1A1. Triplicate measurements were obtained for every tested parameter.

The β-actin (380624), ATF4 (381426), ATF6 (162665), APMAP (860886), FN1 (R381177), N-cadherin (380671), cyclin D1 (CCND1) (380999), Smad2 (R25742), Smad3 (R25743), p-Smad2 (310079), p-Smad3 (R22919), STAT3 (R22785), p-STAT3 (381552), AKT (R23412), p-AKT (310021), p-TAK1 (252059), TAK1 (R382046), p-NFκB p65 (310013), ATP1A1(R380790) antibodies, and the secondary antibodies (511103 and 511203) were obtained from Zenbio. Antibodies against APMAP (25953-1-AP, Proteintech, Wuhan, China), ZEB2 (67514-1-Ig, Proteintech, Wuhan, China), XBP1s (647501, Biolegend, San Diego, CA, USA), TGFBR1 (ab235578, Abcam, Cambridge, UK), CRELD2 (sc-365168, Santa Cruz Biotechnology, Dallas, Texas, USA), TAB2 (A9867, ABclonal Technology, Wuhan, China), TAB1 (RT1603, HuaBio, Hangzhou, China) were also respectively used. Rabbit IgG (KTD105-CN) antibody was obtained from Abbkine. The β-catenin (PK02151) antibody and the specific secondary antibody used to avoid the influence of heavy/light chain (M21008F) were obtained from Abmart (Shanghai, China). All the antibodies were diluted according to the manufacturer’s instructions.

### 2.7 Cell proliferation assay

Cell proliferation ability was assessed using MTS and colony formation assays. For the MTS assay, the 1 × 10^3^ cells were inoculated in 96-well plates. The Cell Titer 96^®^ Aqueous One Solution Cell Proliferation Assay Kit (20 µL) (Promega) was added to the culture medium at 0, 24, 48, 72, and 96 h and incubated for 2 h. The absorbance of each well was detected at 490 nm. For the colony formation assay, the 5 × 10^3^ treated cells were seeded in triplicate in 6-well plates and routinely cultured for approximately 7 days. The colonies were then fixed with 4% paraformaldehyde and stained with 0.1% crystal violet. Colonies composed of 50 cells or more were manually counted.

### 2.8 Cell migration and invasion assays

Cell migration ability was measured by wound healing assay. The treated cells were inoculated into 6-well plates. A scratch wound was created with a sterile 200 µL pipette tip when the cells had grown to 80% confluence, followed by overnight starvation in serum-free medium. The cells were monitored by capturing images at the same fields for 0 and 24 h using an inverted microscope. The percentage of the real-time scratched area was quantified using Image J software.

Invasion assays were conducted using transwell plates (8 µm; Corning Costar, Corning, NY, USA). Briefly, the 1 × 10^5^ transfected cells in 200 µL of RPMI 1640 medium were seeded into the upper compartments of transwells precoated with Matrigel (BD Biosciences, San Jose, CA), while 600 µL of medium supplemented with 10% FBS was added to the lower compartments of the chambers. After 24 h of culture, the cells in the upper chamber were removed, and those on the lower surface of the chamber were fixed with 4% formaldehyde and stained with 0.1% crystal violet. The cells that traversed the membrane were counted under light microscopy at a minimum of 5 predetermined positions per membrane.

### 2.9 Immunofluorescence

The treated KYSE150 cells were seeded on the coverslips (NEST, Wuxi, China) at the appropriate density. The cells were fixed in 4% paraformaldehyde for 20 min and permeabilized with 0.1% Triton X-100 for 15 min. After washing with PBS, the cells were blocked with 2% bovine serum albumin, and then incubated with the rabbit anti-human polyclonal antibody APMAP (Cusabio, Wuhan, China, dilution at 1:100) at 4°C for 24 h. Finally, the cells were incubated with a fluorescent secondary antibody for 1 h at room temperature. Nuclei were stained with DAPI. The protein labeling was visualized under a confocal microscope.

### 2.10 Flow cytometry analysis of cell cycle

Cell cycle distribution was analyzed by propidium iodide (PI; Multi Sciences, Hangzhou, China) mono-staining. Post-transfection cells were collected and resuspended to a concentration of 1×10^6^ cells/mL. After being washed twice with ice-cold PBS, cell suspensions were incubated with 500 μL of PI working solution for 30 minutes at 4°C in the dark. Cell cycle profiles were subsequently acquired using a FC-500 type flow cytometer (Beckman, Pasadena, USA).

### 2.11 Statistical analysis

Statistical analysis and graphing were conducted using GraphPad Prism 8.0 (La Jolla, CA, USA). The quantitative data, derived from at least three independent biological replicates. Comparisons between two groups were performed using Student’s t-test, while multiple group comparisons were analyzed by one-way ANOVA analysis. Data analysis was presented as mean ± standard deviation (S.D). Statistical significance was defined as *p < 0.05, ** p< 0.01.

## 3 Results

### 3.1 ER stress induces upregulation of CRELD2 in ESCC cells

**Figure 1:**
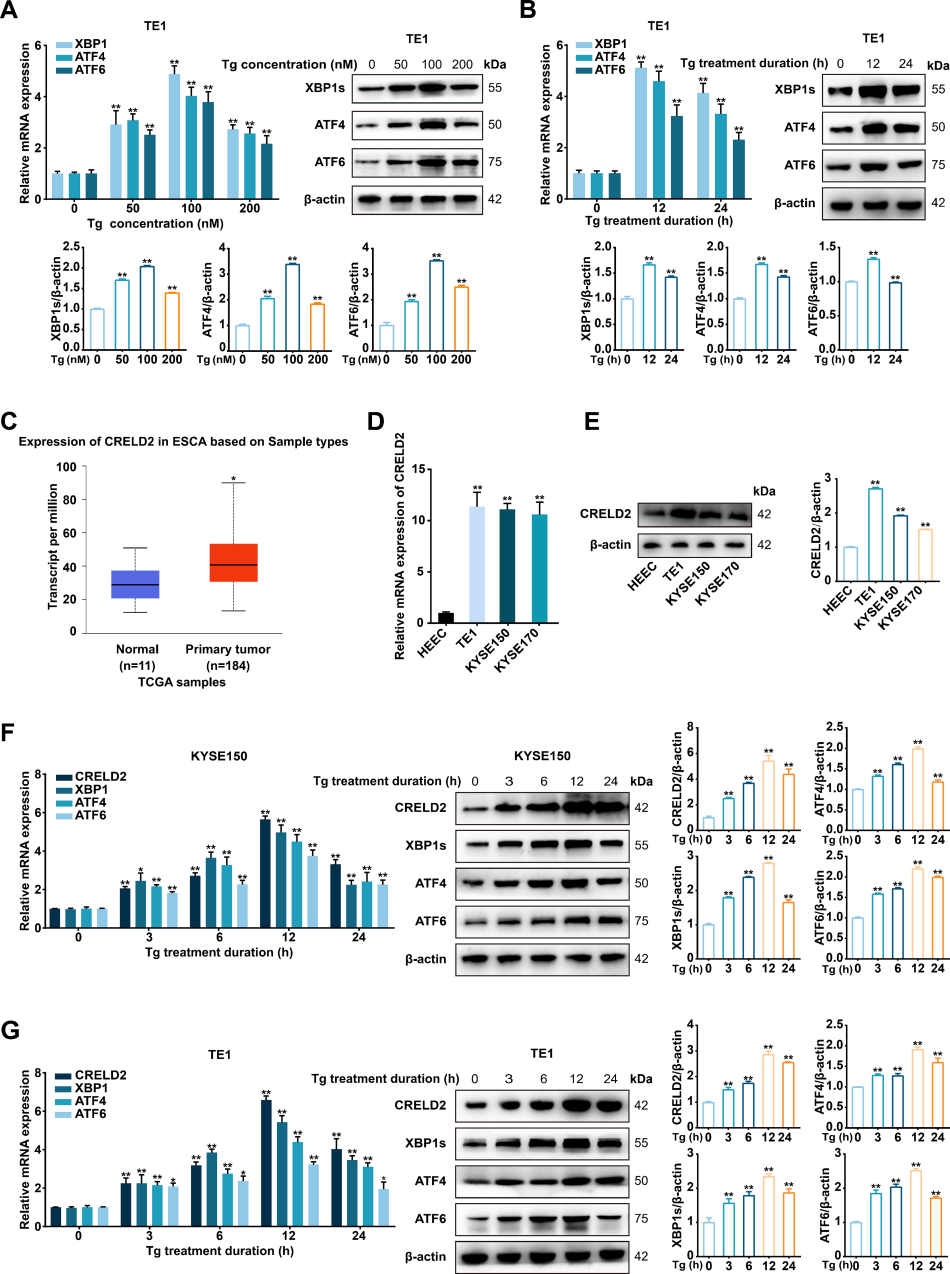
Endoplasmic reticulum stress modulates CRELD2 expression in ESCC cells. **(A)** qRT-PCR and Western blot analysis of XBP1, ATF4, and ATF6 expression in TE1 cells treated with thapsigargin (Tg) at concentrations of 0, 50, 100, and 200 nM. **(B)** qRT-PCR and Western blot analysis of TE1 cells following treated with 100 nM Tg for 0, 12, and 24 h. **(C)** The expression profile of CRELD2 was systematically analyzed in tumor tissues and paired normal counterparts using the UALCAN database. **(D, E)** qRT-PCR **(D)** and Western blot **(E)** analysis of CRELD2 expression in human normal esophageal epithelial cells (HEEC) and ESCC cell lines (TE1, KYSE150, and KYSE170). **(F, G)** qRT-PCR and Western blot analysis of CRELD2 expression in KYSE150 **(F)** and TE1 **(G)** cells treated with 100 nM Tg for the indicated treatment durations. Tg (nM), Tg concentration (nM). Tg (h), Tg treatment duration (h). The protein levels were quantified by band densitometry. Data represent the mean ± SD of three independent experiments. *P < 0.05, **P < 0.01.

To investigate the appropriate conditions for inducing ER stress in ESCC cells *in vitro*, we selected Tg as an inducer. Initially, TE1 cells were respectively treated with 50 nM, 100 nM, and 200 nM Tg for 12 h. The mRNA and protein expression levels of UPR effectors XBP1, ATF4, and ATF6 were notably increased, particularly at 100 nM (**Figure 1A**), thus 100 nM Tg was determined as the optimal concentration. TE1 cells were further incubated with 100 nM Tg for 12 h and 24 h, and the alterations in the UPR marker expression levels indicated that 12 h was the optimal treatment duration (**Figure 1B**). To ascertain the potential role of ER stress in ESCC cells, we performed RNA-sequencing analysis on TE1 cells treated with 100 nM Tg for 12 h, using DMSO-treated cells as a control (15), and CRELD2 was one of the most significantly differentially expressed genes. Further analysis of the UALCAN database (http://ualcan.path.uab.edu) revealed significant upregulation of CRELD2 in esophageal cancer (**Figure 1C**). The increased mRNA and protein expression levels of CRELD2 were also detected in three ESCC cell lines compared with normal esophageal epithelial cells (HEEC) (**Figures 1D, E**). Moreover, the upregulation of CRELD2 at mRNA and protein expression levels in response to ER stress was confirmed in both KYSE150 and TE1 cells, correlating with the expression of XBP1, ATF4, and ATF6 (**Figures 1F, G**). Given the biphasic nature of ER stress responses in tumor cells, we performed extended Tg treatment time-course (0-96 h) experiments in KYSE150 and TE1 cells. The expression level of CRELD2 peaked at 12 hours and subsequently declined during the late ER stress phase (48-96 hours) (**Supplementary Figure S1A**). This phase-specific expression pattern suggests that CRELD2 may act as an early ER stress-inducible gene, primarily responsible for regulating early-phase ER stress response in ESCC cells.

### 3.2 The PERK/ATF4 pathway contributes to induction of CRELD2

**Figure 2:**
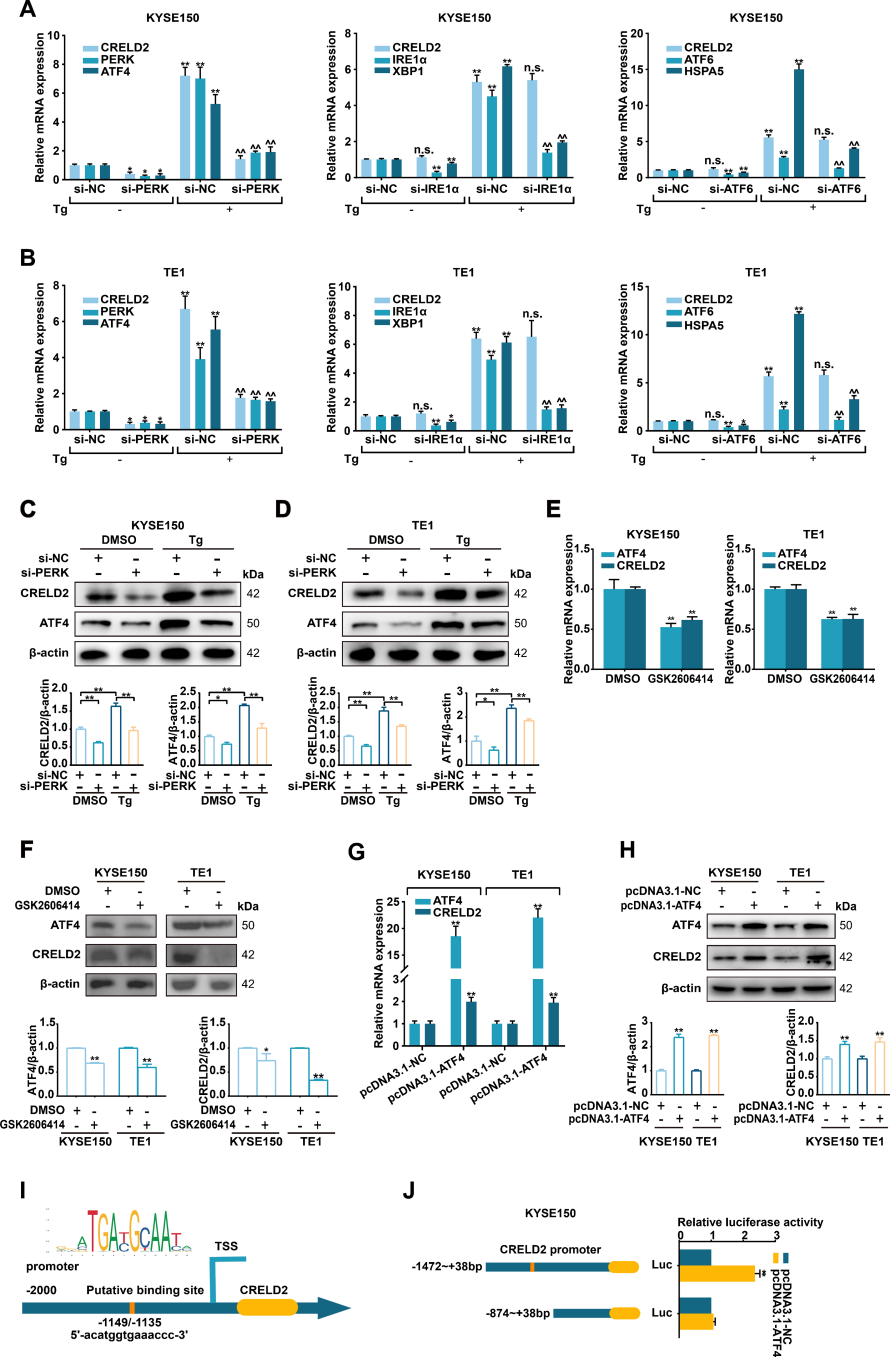
CRELD2 is induced by the PERK pathway of the UPR in ESCC cells. **(A, B)** qRT-PCR analysis of the given mRNA levels in KYSE150 **(A)** and TE1 **(B)** cells transfected with si-NC, si-PERK, si-IRE1α or si-ATF6, and treated with 100 nM Tg (+) or DMSO (−) for 12 h. **(C, D)** Western blot analysis of siRNA-transfected KYSE150 **(C)** and TE1 **(D)** cells treated with 100 nM Tg or DMSO for 12 h. **(E, F)** qRT-PCR **(E)** and Western blot **(F)** analysis of ATF4 and CRELD2 expression in KYSE150 and TE1 cells treated with DMSO or 2 µM GSK2606414 for 48 h. **(G, H)** qRT-PCR **(G)** and Western blot **(H)** assays of the indicated genes in ATF4-overexpressing KYSE150 and TE1 cells. **(I)** The probable ATF4 binding site in the promoter region of CRELD2. **(J)** Luciferase reporter assays were performed by cotransfecting KYSE150 cells with the CRELD2 promoter fragment and the ATF4 overexpression construct. The protein levels were quantified by band densitometry. Data represent the mean ± SD of three independent experiments. *P < 0.05, **P < 0.01 for comparisons to si-NC or pcDNA3.1-NC *without Tg or ^with Tg treatment. n.s., not significant.

To recognize which branch of the UPR contributes to CRELD2 induction, as depicted in **Figures 2A, B**, three siRNAs (targeting PERK, IRE1α, and ATF6) were employed to individually silence the three ER transmembrane sensors in KYSE150 and TE1 cells. The depletion of PERK not only downregulated its canonical target ATF4 but also concomitantly impaired Tg-induced CRELD2 upregulation at both mRNA and protein expression levels (**Figures 2A–D**). Consistent with PERK pathway involvement, GSK2606414 (a PERK kinase inhibitor)-mediated blockade significantly decreased both mRNA and protein expression levels of ATF4, with a corresponding reduction in CRELD2 expression levels in KYSE150 and TE1 cells (**Figures 2E, F**). In contrast, the knockdown of IRE1α and ATF6 disrupted the induction of XBP1 and HSPA5 but not CRELD2 (**Figures 2A, B**). ATF4, a canonical target of PERK, plays a critical role as a transcription factor in ER stress. To explore the role of ATF4 in regulating transcription and expression of CRELD2, we initially overexpressed ATF4 in KYSE150 and TE1 cells, which was sufficient to increase the mRNA and protein expression levels of CRELD2 (**Figures 2G, H**). A putative ATF4 binding site in CRELD2 promoter region (-1149 ~ -1135 bp) was further predicted using JASPAR database (**Figure 2I**), and luciferase assays showed that ATF4-mediated activation of the CRELD2 promoter reporter construct was significantly dependent on this region (**Figure 2J**). These findings demonstrate that PERK/ATF4 pathway promotes transcription and expression of CRELD2 in response to ER stress in ESCC cells.

### 3.3 CRELD2 promotes ESCC cells proliferation, migration, and invasion *in vitro*

**Figure 3:**
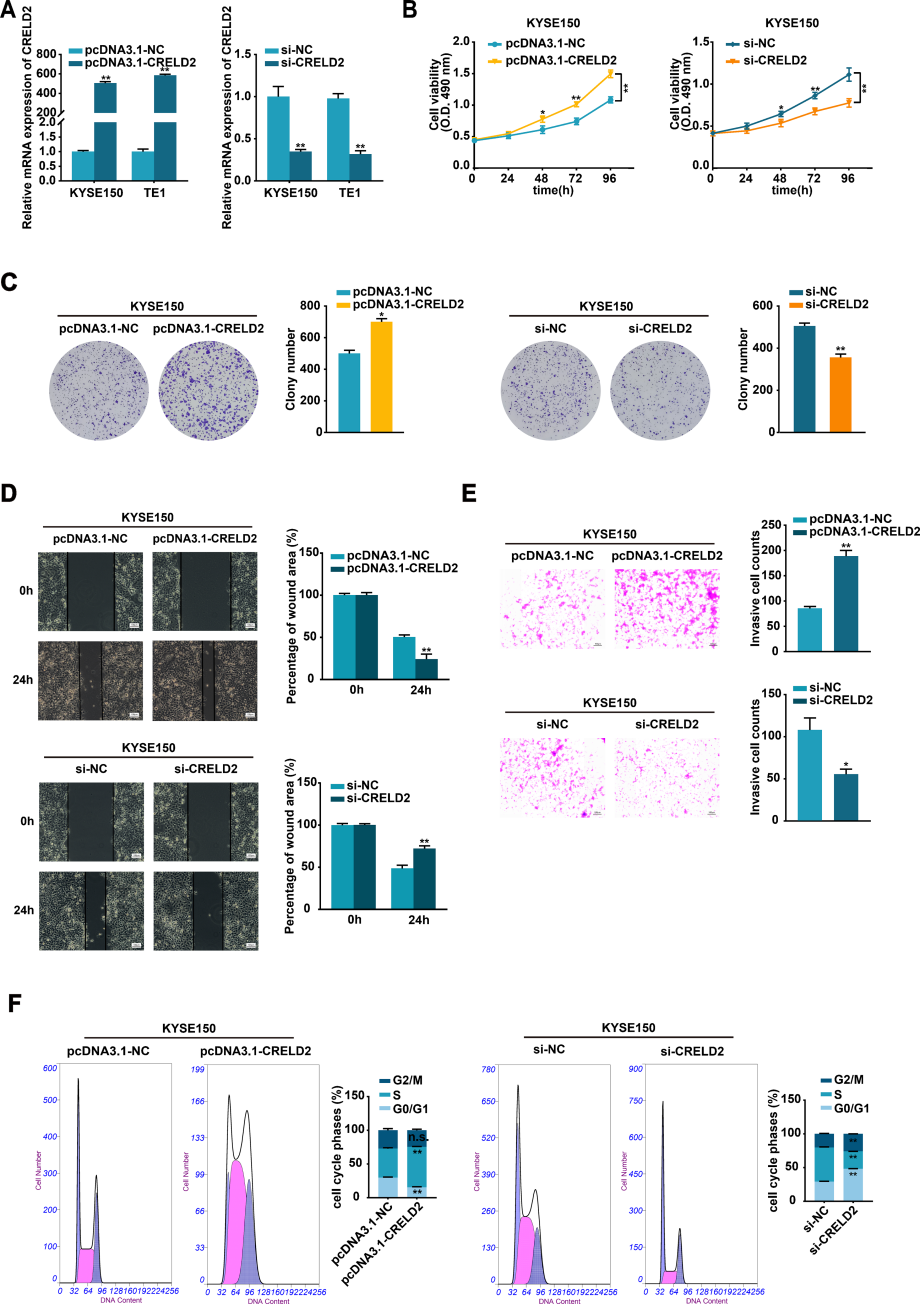
CRELD2 promotes the proliferation, migration, and invasion of ESCC cells. **(A)** The transfection efficiency of CRELD2 overexpression and knockdown in KYSE150 and TE1 cells was assessed using the qRT-PCR method. **(B, C)** MTS **(B)** and colony formation **(C)** assays showed enhanced cell viability and clonogenic potential in CRELD2-overexpressing KYSE150 and TE1 cells compared to controls. Corresponding knockdown experiments produced opposing effects. **(D, E)** CRELD2 overexpression accelerated both cell migration (wound healing assay) **(D)** and invasive (transwell assay) **(E)**, whereas its knockdown showed inhibitory effects. Scale bar, 100 μm. **(F)** Flow cytometric cell cycle analysis of KYSE150 cells with CRELD2 overexpression or knockdown. Data represent the mean ± SD of three independent experiments. A representative data from three independent experiments is shown. *P < 0.05, **P < 0.01, n.s., not significant.

To investigate the potential effects of CRELD2 on the malignant biological behavior of ESCC cells *in vitro*, the pcDNA3.1-CRELD2 plasmid and si-CRELD2 were respectively employed to upregulate or downregulate the expression of CRELD2 in KYSE150 and TE1 cells (**Figure 3A**). Overexpression of CRELD2 markedly increased viability and colony-forming ability of KYSE150 and TE1 cells detected by MTS and colony formation assays (**Figures 3B, C**; **Supplementary Figures S2A, B**). Moreover, overexpression of CRELD2 promoted KYSE150 and TE1 cells migration and invasion, as evidenced by wound healing and Transwell assays (**Figures 3D, E**; **Supplementary Figures S2C, D**). Conversely, knockdown of CRELD2 produced the opposite effects (**Figures 3B–E**; **Supplementary Figures S2A–D**). Flow cytometric cell cycle analysis revealed increased S-phase entry in CRELD2-overexpressing cells, whereas knockdown of CRELD2 caused G1 phase accumulation (**Figure 3F**; **Supplementary Figure S2E**). Collectively, our findings demonstrate that CRELD2 may drive malignant phenotypes in ESCC cells *in vitro*.

### 3.4 CRELD2 mediates the ER stress-regulated malignant biological behavior in ESCC cells

**Figure 4:**
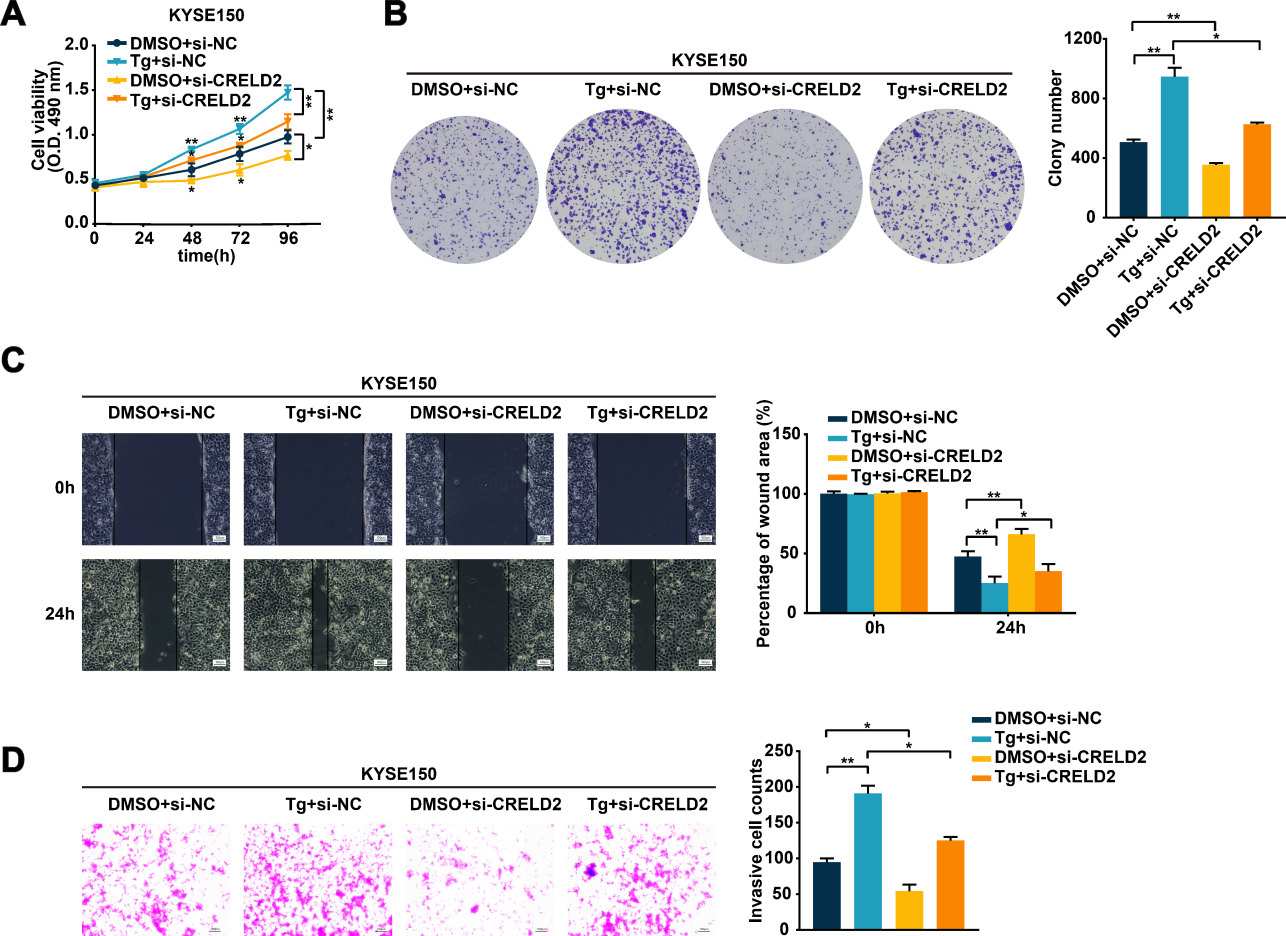
CRELD2 mediates the ERS-regulated malignant biological behavior in ESCC cells. **(A, B)** Cell proliferation ability was assessed by MTS **(A)** and colony formation **(B)** assays in the KYSE150 and TE1 cells transfected with si-NC or si-CRELD2 and treated with 100 nM Tg or DMSO for 12 h. **(C, D)** Wound healing **(C)** and transwell **(D)** assays were conducted to evaluate the migration and invasion abilities of KYSE150 and TE1 cells transfected with si-NC or si-CRELD2 and treated with Tg (100 nM) or DMSO for 12 h. Scale bar, 100 μm. Data represent the mean ± SD of three independent experiments. A representative data from three independent experiments is shown. *P < 0.05, **P < 0.01.

Given the evident oncogenic role of CRELD2, we further performed functional assays to determine whether CRELD2 mediates ER stress-regulated ESCC progression. Knockdown of CRELD2 significantly attenuated the Tg-induced proliferation of KYSE150 and TE1 cells (**Figures 4A, B**; **Supplementary Figures S3A, B**). Likewise, the enhanced migration and invasion abilities of KYSE150 and TE1 cells resulting from Tg treatment were also diminished by the knockdown of CRELD2 (**Figures 4C, D**; **Supplementary Figures S3C, D**). These findings demonstrate that CRELD2 is critical for ER stress-driven malignant progression in ESCC.

### 3.5 CRELD2 increases the membrane localization of APMAP

**Figure 5:**
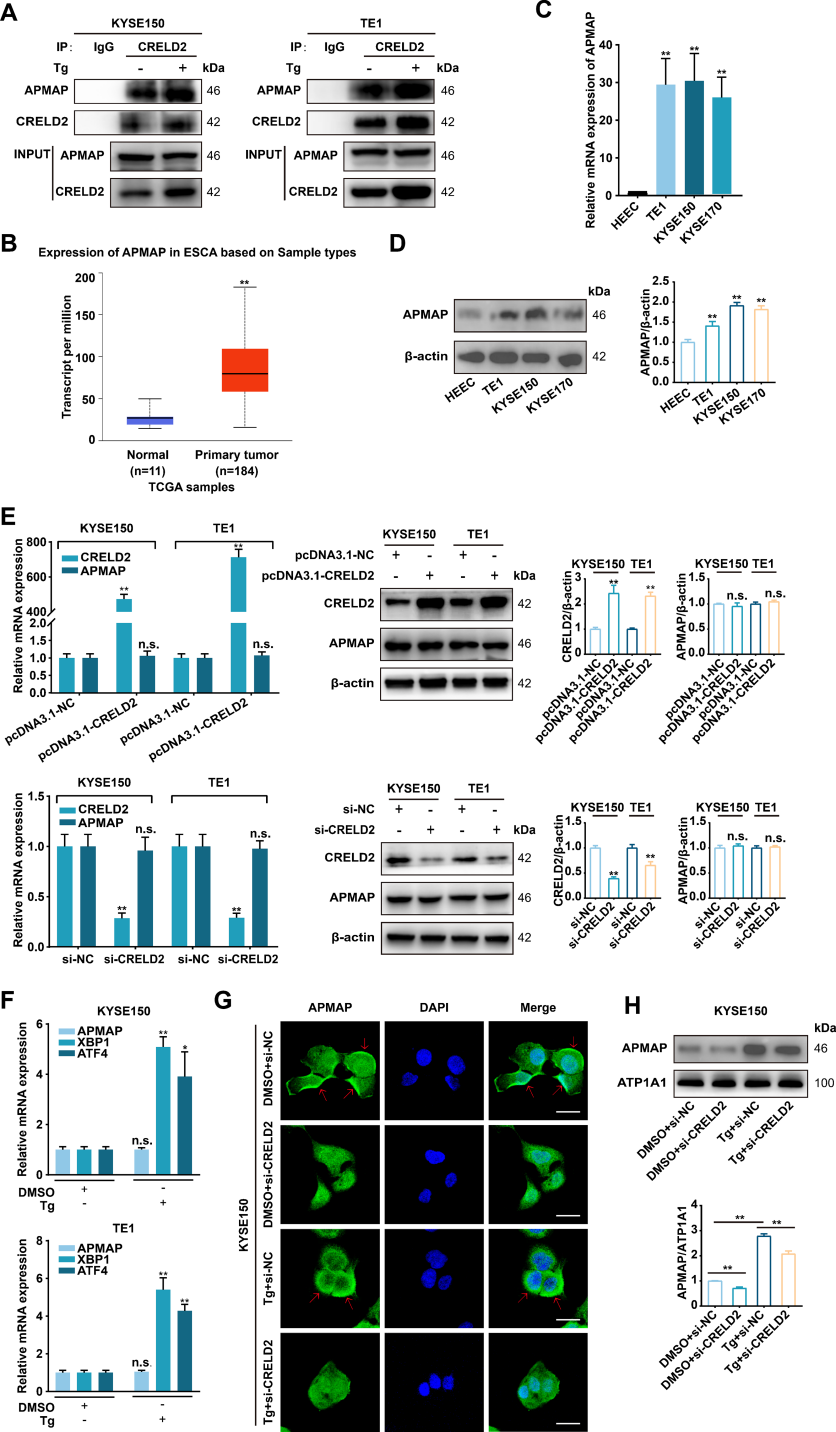
CRELD2 increases the membrane localization of APMAP. **(A)** The interaction between CRELD2 and APMAP was detected in the indicated cells by co-immunoprecipitation assay using anti-CRELD2 followed by immunoblot with anti-APMAP or anti-CRELD2. **(B)** The expression profile of APMAP was systematically analyzed in tumor tissues and paired normal counterparts using the UALCAN database. **(C, D)** qRT-PCR **(C)** and Western blot **(D)** analysis of APMAP expression in HEEC and ESCC cell lines (TE1, KYSE150, and KYSE170). **(E)** qRT-PCR and Western blot analysis of CRELD2 and APMAP expression in TE1 and KYSE150 cells with CRELD2 overexpression or knockdown. **(F)** qRT-PCR assay of KYSE150 and TE1 cells treated with Tg (+) or DMSO (–) for 12 h. **(G)** The subcellular localization of APMAP in KYSE150 cells treated as indicated was measured by immunofluorescence staining. Nuclei are stained with DAPI. Arrows indicate plasma membrane localization of APMAP. Scale bar, 25 μm. **(H)** Western blot showing APMAP expression in the membrane fraction from each treatment condition. The protein levels were quantified by band densitometry. Data represent the mean ± SD of three independent experiments. *P < 0.05, **P < 0.01, n.s., not significant.

We subsequently explored the underlying mechanism of CRELD2 in ESCC progression. Current studies support that CRELD2 is not only an endoplasmic reticulum-resident protein but also a secreted protein (10), and the subcellular localization of CRELD2 suggests a potential role for CRELD2 in trafficking proteins from the ER (16). The CRELD2 binding chaperones were identified via LC-MS/MS assay in chondrocytes and osteoblasts (16), and among the publicly reported potential binding proteins, adipocyte plasma membrane-associated protein (APMAP) caught our attention due to its localization and evident function. The physical interaction of CRELD2 with APMAP was verified by co-immunoprecipitation (CO-IP) assay in KYSE150 and TE1 cells, and the binding effect was further enhanced in Tg-treated cells (**Figure 5A**). Further analysis of the UALCAN database revealed significant upregulation of APMAP in esophageal cancer (**Figure 5B**), and the increased mRNA and protein expression levels of APMAP were also detected in three ESCC cell lines compared with HEEC cells (**Figures 5C, D**).

We further investigated the functional interplay between CRELD2 and APMAP. As shown in **Figure 5E**, neither overexpression nor knockdown of CRELD2 significantly altered mRNA or protein expression levels of APMAP in KYSE150 and TE1 cells. Moreover, the mRNA expression level of APMAP was not affected by Tg treatment (**Figure 5F**). Immunofluorescence was then employed to investigate whether CRELD2 could influence the location of APMAP, and as shown in **Figure 5G**, APMAP was located mainly on the cell membrane, and the fluorescence of APMAP on the membrane was significantly increased in Tg-treated cells, whereas this effect was partially blocked by the knockdown of CRELD2. Western blot analysis of membrane fractions revealed significantly increased APMAP localization at the plasma membrane following Tg treatment, which was partially reversed in CRELD2-deficient cells (**Figure 5H**). These data suggest that CRELD2 may act as a molecular chaperone for APMAP and promote APMAP localization to the cell membrane under ER stress condition.

### 3.6 APMAP silencing alleviates the malignant phenotype of ESCC cells *in vitro*

**Figure 6:**
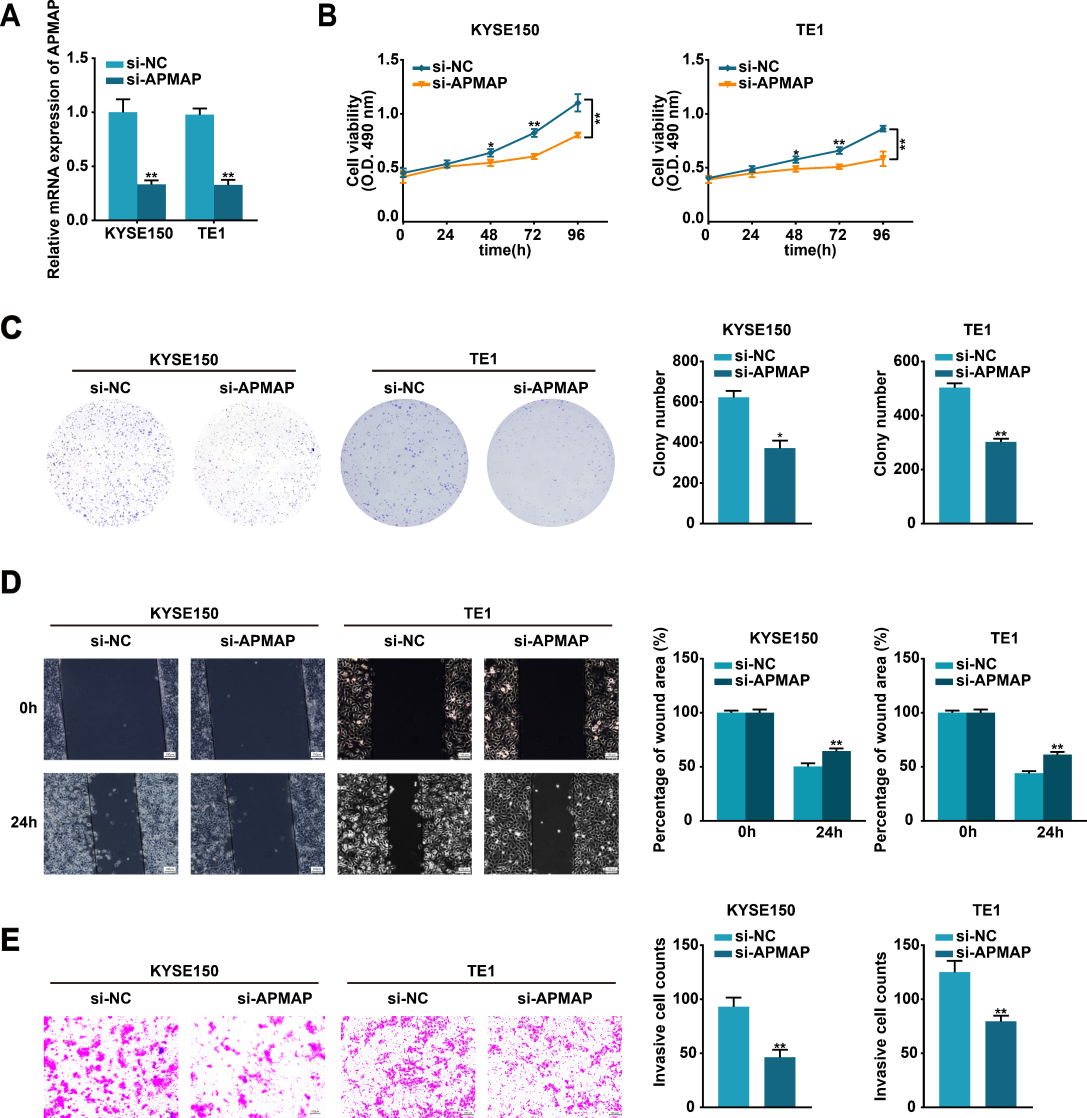
Silencing of APMAP alleviates the malignant phenotype of ESCC cells. **(A)** The transfection efficiency of si-APMAP in KYSE150 and TE1 cells was detected by qRT-PCR method. **(B, C)** The proliferation ability of the indicated cells was assessed by MTS **(B)** and colony formation **(C)** assays. **(D, E)** Cell migration and invasion of the indicated cells were verified through wound healing **(D)** and transwell invasion **(E)** assays. Scale bar, 100 μm. Data represent the mean ± SD of three independent experiments. *P < 0.05, **P < 0.01.

To further investigate the biological function of APMAP in ESCC cells, si-APMAP was used to knock down the expression of APMAP in KYSE150 and TE1 cells and the knockdown efficiency was verified using qRT-PCR method (**Figure 6A**). The knockdown of APMAP impaired the proliferation, migration, and invasion abilities of KYSE150 and TE1 cells, as detected by MTS, colony formation, wound healing, and transwell assays (**Figures 6B–E**). In summary, APMAP may function as a cancerous gene in ESCC.

### 3.7 APMAP exerts carcinogenic effects through activation of TGF-β/SMAD and NF-κB pathways

**Figure 7:**
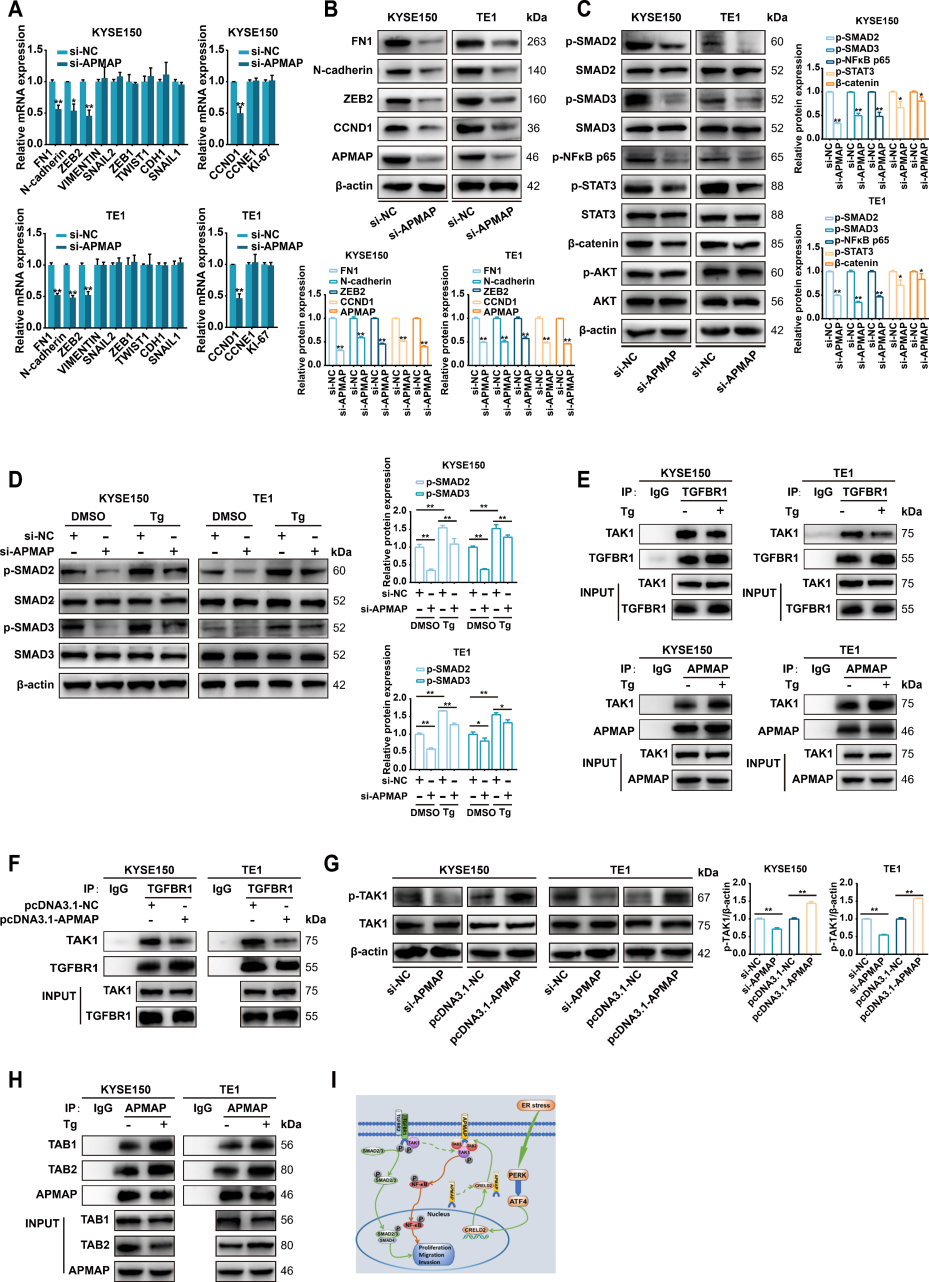
APMAP activates TGF-β/SMAD and NF-κB signaling during ER stress-induced EMT and proliferation. **(A)** qRT-PCR analysis of EMT and proliferation marker expression in KYSE150 and TE1 cells transfected with control siRNA (si-NC) or si-APMAP. **(B)** Western blot analysis of the indicated proteins expression in KYSE150 and TE1 cells transfected with si-NC or si-APMAP. **(C)** The protein expression of key genes of the TGF-β/SMAD, NF-κB, IL6/STAT3, Wnt/β-catenin, and PI3K/AKT signaling pathways in APMAP-knockdown KYSE150 and TE1 cells was examined by Western blot assay. **(D)** The effect of APMAP knockdown on the protein expression of SMAD2/3 and p-SMAD2/3 in Tg (100 nM, 12 h) treated KYSE150 and TE1 cells. **(E, F)** Co-immunoprecipitation assay was conducted with anti-TGFBR1 or anti-APMAP antibodies followed by immunoblot with anti-TAK1, anti-TGFBR1 or anti-APMAP in the indicated cells. **(G)** Western blot analysis of p-TAK1 and TAK1 expression in APMAP-manipulated KYSE150 cells and TE1 cells (overexpression or knockdown). **(H)** Co-immunoprecipitation assay was conducted with anti-APMAP antibody followed by immunoblot with anti-TAB1, anti-TAB2, or anti-APMAP in the indicated cells. **(I)** The proposed model for ER stress-induced CRELD2 promotes membrane the localization of APMAP, which increases its interaction with TAK1, TAB1, and TAB2, thereby promoting the activation of the TGF-β/SMAD and NF-κB pathways and inducing the EMT and proliferation of ESCC cells, is shown. The protein levels were quantified by band densitometry. Data represent the mean ± SD of three independent experiments. *P < 0.05, **P < 0.01.

Since the ER stress-inducible CRELD2 can increase the membrane localization of APMAP, and both genes exhibit significant oncogenic effects, we further explored the potential mechanisms of APMAP in ESCC. Given the critical role of epithelial-mesenchymal transition (EMT) in cell migration and invasion (17), we systematically evaluated the impact of APMAP on key EMT and proliferation markers, including FN1, N-cadherin, ZEB2, VIMENTIN, SNAIL2, ZEB1, TWIST1, CDH1, SNAIL1, CCND1, CCNE1, and KI-67 (**Figure 7A**). Knockdown of APMAP in KYSE150 and TE1 cells consistently downregulated expression of FN1, N-cadherin, ZEB2, and CCND1 at both mRNA (**Figure 7A**) and protein levels (**Figure 7B**). These results suggest that APMAP may promote migration and invasion of ESCC by regulating EMT and enhances proliferation through regulating CCND1. Furthermore, overexpression or knockdown of CRELD2 in KYSE150 and TE1 cells recapitulated corresponding expression changes in APMAP-regulated genes (**Supplementary Figures S4A, B**). Given the established role of CRELD2 in promoting APMAP membrane localization, our findings collectively suggest that APMAP may act as the downstream executor of CRELD2-mediated signaling, transducing upstream stress responses into the regulation of EMT and proliferation.

To further investigate the signaling pathways underlying APMAP-mediated EMT and proliferation, we analyzed the effect of APMAP knockdown on key proteins in EMT- and proliferation-related pathways, including the TGF-β/SMAD, NF-κB, IL6/STAT3, Wnt/β-catenin, and PI3K/AKT signaling pathways (**Figure 7C**). Markedly decreased phosphorylation of SMAD2/3 and NF-κB p65 was observed upon APMAP knockdown in both KYSE150 and TE1 cell, whereas no significant reduction in STAT3 or β-catenin phosphorylation was detected, and AKT phosphorylation remained unaffected (**Figure 7C**). These results suggest that APMAP may promote carcinogenesis primarily through activation of TGF-β/SMAD and NF-κB pathway.

### 3.8 APMAP mediates TGF-β/SMAD activation by binding to TAK1 in competition with TGFBR1 under ER stress condition

We further investigated the activation of p-SMAD2/3 and the regulatory role of APMAP under ER stress. As shown in **Figure 7D**, upregulation of p-SMAD2/3 was observed in Tg-treated KYSE150 and TE1 cells, which was partially inhibited by APMAP knockdown, indicating that the TGF-β/SMAD signaling pathway was activated under ER stress and that APMAP was involved in this effect. Given that the binding of CRELD2 to APMAP promotes APMAP localization on the cell membrane under ER stress, we speculated whether APMAP might be involved in regulating the activation or release of TGF-β receptors (TGFBRs) which are also localized on the cell membrane. It has been reported that endogenous TAK1 stably coimmunoprecipitates with TGFBR1 under TGF-β1 unstimulated condition, and TGF-β1 stimulation triggers the dissociation of TAK1 from TGFBR1 and induces phosphorylation and activation of TGFBR1 (18). We speculated that whether APMAP could competitively bind with TAK1 under ER stress and subsequently lead to the dissociation of TAK1 from TGFBR1 to further activate the TGF-β/SMAD signaling pathway. As provided in **Figure 7E**, endogenous TAK1 was coimmunoprecipitated with TGFBR1 both in KYSE150 and TE1 cells, while Tg treatment attenuated this binding effect. Conversely, the association of APMAP with TAK1 was significantly increased in Tg-treated ESCC cells. An APMAP overexpression plasmid was further constructed (**Supplementary Figures S5A, B**), and the overexpression of APMAP significantly reduced the interaction of TAK1 with TGFBR1 (**Figure 7F**), indicating that APMAP may mediate TGF-β/SMAD activation by binding to TAK1 in competition with TGFBR1 under ER stress condition.

### 3.9 APMAP mediates activation of the NF-κB pathway by contributing to the formation of TAK1, TAB1, and TAB2 complexes to promote TAK1 phosphorylation

We then investigated the regulatory role of APMAP in the activation of the NF-κB pathway. Phosphorylation-activated TAK1 can transduce signals to several downstream signaling cascades, including the MKK3/6-p38 MAPK cascade, MAPK kinase (MKK) 4/7-JNK cascade, and NF-κB inducing kinase-IκB kinase cascade (19). We speculated that in addition to binding to TAK1, APMAP may promote its phosphorylation and activation, thereby activating the NF-κB pathway. As shown in **Figure 7G**, knockdown of APMAP attenuated TAK1 phosphorylation, while overexpression of APMAP promoted the phosphorylation of TAK1, suggesting the regulatory role of APMAP in TAK1 phosphorylation and activation. However, APMAP does not possess kinase activity; we therefore conjectured that APMAP may indirectly regulate the phosphorylation of TAK1. It has been demonstrated that TAK1 can be activated by its interaction with TAK1-binding protein 1 (TAB1) and TAK1-binding protein 2 (TAB2). TAK1 forms complexes with TAB1 and TAB2, which contribute to the autophosphorylation of TAK1 (20). The physical interaction of APMAP with TAB1 and TAB2 was verified in KYSE150 and TE1 cells, and this binding effect was enhanced by Tg treatment (**Figure 7H**). These data suggest that APMAP may regulate activation of NF-κB pathway through promoting the phosphorylation of TAK1 by contributing to the formation of ternal complexes of TAK1, TAB1, and TAB2 under ER stress condition.

## 4 Discussion

ER stress signaling is relevant to all stages of cancer development and holds promise as a valuable new therapeutic target for treating a wide range of cancers (21). Recent studies have reported that ER stress also regulates the malignant phenotype of ESCC cells. For example, the activation of the IRE1/JNK pathway by GPx8 increased proliferation and inhibited apoptosis in ESCC (22). XBP1-induced MMP-9 promoted proliferation and invasion in ESCC (23). ER stress-inducible TMTC3 markedly increased tumor angiogenesis through the activation of the Rho GTPase/STAT3 pathway in ESCC (24). However, the exact function and underlying mechanism of ER stress in ESCC still require further exploration. In this study, we revealed that CRELD2 was a pivotal ER stress-inducible gene in ESCC that might be regulated by the PERK/ATF4 pathway of the UPR. CRELD2 partially mediated ER stress-stimulated proliferation, migration, and invasion capacity of ESCC cells. Additionally, CRELD2 increased the membrane localization of APMAP, which subsequently activated the TGF-β/SMAD pathway by competing with TGFBR1 for binding to TAK1 and activated the NF-κB pathway by promoting the phosphorylation of TAK1 through its contribution to the formation of ternary complexes of TAK1, TAB1, and TAB2. These findings indicate that APMAP may act as the downstream executor of CRELD2-mediated signaling, transducing upstream ER stress responses into the regulation of EMT and proliferation.

CRELD2, originally identified as the second member of the CRELD protein family (25), is both an endoplasmic reticulum-resident protein and a secreted protein, and its functions are relevant mainly to protein folding and transport (10). CRELD2 exhibits protein disulfide isomerase (PDI)-like activity in complex ER stress responses, which is essential for correct disulfide bond formation and remodeling during the UPR (26). In the present study, we found that CRELD2 was one of the ER stress-induced genes with significantly increased expression in ESCC cells, and it was an early inducible gene whose high expression was regulated by the PERK-ATF4 pathway. Consistently, CRELD2 has been shown to be transcriptionally regulated by ATF4 in breast cancer (12). Additionally, it has been shown that CRELD2 can also be regulated by ATF6 (27, 28), suggesting the heterogeneity of the transcriptional regulation of ERS-stimulated CRELD2 across different cellular contexts, which needs to be further investigated in different tumors afterwards. CRELD2 has been implicated in various physiological and pathological processes, including hepatic metabolic homeostasis, cartilage and bone metabolism, and cancer progression (12, 13, 29, 30). However, the role of CRELD2 in the carcinogenesis of ESCC remains largely unknown. Our study evaluated that CRELD2, as an ER stress-inducible gene, promoted the proliferation, migration, and invasion of ESCC cells *in vitro* and mediated ER stress-induced malignant biological behaviors in ESCC cells, thus acting as an oncogene.

In the present study, we demonstrated that CRELD2 co-immunoprecipitates with APMAP without altering its total protein expression levels. However, CRELD2 significantly enhanced membrane localization of APMAP, as confirmed by both subcellular fractionation and immunofluorescence assays, which suggests that CRELD2 may play a significant role by binding to APMAP and subsequently influencing its localization. This effect may be attributed to the presence of a KDEL-like ER retention sequence (REDL) in CRELD2. By searching the UniProt database, only four other mammalian proteins with an REDL motif were identified. Two of these proteins, CNPY4 and MESDC2, function as chaperones and enhance the folding, maturation, or cell surface expression of receptors (31, 32). We proposed that CRELD2 may also act as a molecular chaperone to facilitate correct APMAP folding, maintain its conformational stability, protect N-terminal signal peptide integrity for recognition, and ultimately promote APMAP membrane localization. Consistently, CRELD2 has been reported to function as a protein chaperone for low-density lipoprotein receptor-related protein 1 (LRP1) and TGF-β1, facilitating their trafficking and secretion (16, 30).

APMAP, also known as C20orf3, is an integral glycosyl-type II plasma membrane protein (33). Previous studies have mainly focused on the association of APMAP with adipose differentiation (34). More recently, it has been identified as an oncogenic driver in various cancers (35, 36). The APMAP/EGFR axis enhances cholesterol-induced EMT in prostatic carcinoma (35), and APMAP promotes metastasis of cervical cancer by activating the Wnt/β-catenin pathway (36). However, the functional roles and molecular mechanisms of APMAP remains poorly understood in cancer research, particularly in ESCC. Our study revealed the oncogenic role of APMAP in ESCC by promoting the malignant biological behavior and EMT process of ESCC cells.

APMAP is characterized as a transmembrane protein and can induce EMT in prostate cancer and liver metastasis in colorectal cancer (35, 37, 38). Cholesterol facilitates the competitive binding of APMAP and EGFR substrate 15-related protein (EPS15R), which in turn increases EGFR stability and activates ERK1/2 to promote EMT (35). In the present study, we found that APMAP competed with TGFBR1 for binding to TAK1, which subsequently led to the dissociation of TAK1 from TGFBR1 to further activate the TGF-β/SMAD pathway. Additionally, it was interesting to observe that APMAP could bind not only to TAK1 but also to TAB1 and TAB2, thereby promoting the formation of the TAK1/TAB1/TAB2 complex, which favors the activation of TAK1 and subsequently activates the NF-κB pathway. TAK1, also known as MAP3K7 or NR2C2, is stably associated with the GS structural domain of TGFBR1 under unstimulated conditions (18), and the activation of TAK1, which depends on TAB1 and TAB2 or its homologous protein TAB3, is critical for the activation of the NF-κB pathway (18–20, 39). TGF-β/SMAD and NF-κB are the major pathways responsible for the activation of EMT during tumorigenesis (40, 41). TGF-β can stimulate FN1 synthesis via c-Jun N-terminal kinase, disabled-2, SOX4, and FOXC2 (42–45). Similarly, TGF-β can modulate the transcription of ZEB2 and N-cadherin (44–46). NF-κB also plays a vital role in the transcription of ZEB2 and N-cadherin (40, 47). Thus, we speculated that APMAP promoted EMT by inducing the activation of TGF-β/SMAD and NF-κB pathways. Since activated NF-κB protein can transcriptionally regulate CCND1 by directly binding to its promoter (48), and CCND1 also serves as a target gene for STAT3 (49), we believed that APMAP interfered the proliferation ability of ESCC cells by modulating the expression of CCND1, and at least in part, through the regulation of the NF-κB pathway.

## 5 Conclusion

In summary, our data provide a new insight into the ER stress-related progression of ESCC. We reveal that the ER stress-inducible CRELD2 enhances the membrane localization of APMAP, which in turn increases its binding to TAK1 during ER stress. This interaction facilitates the activation of the TGF-β/SMAD and NF-κB pathways, inducing EMT and proliferation in ESCC (**Figure 7I**). Our study provides a plausible explanation for the roles of CRELD2 and APMAP in cancer cells undergoing ER stress.

## Data Availability

The data generated in this study have been deposited in the NCBI Sequence Read Archive under project PRJNA1256794 with accession numbers SRR33388730 and SRR33388731.
