# Supplementary material for: Endoplasmic reticulum stress-induced CRELD2 promotes APMAP-mediated activation of TGF-β/SMAD and NF-κB pathways in esophageal squamous cell carcinoma
Source: Front Immunol. 2025 Jul 31;16:1616201. doi: 10.3389/fimmu.2025.1616201 (PMC12351132; doi:10.3389/fimmu.2025.1616201)
Supplement: Supplementary file 1 [file DataSheet1.pdf]

Supplementary Material

Supplementary Tables

Table S1

Primer sequences used for qRT-PCR assays.

Gene	Primer sequence (5'-3')
GAPDH	Forward: GAAGGTGAAGGTCGGAGTC
	Reverse: GAAGATGGTGATGGGATTTC
TWIST1	Forward: ACTTCCTCTACCAGGTCCTCCAG
	Reverse: CCTCCATCCTCCAGACCGAGAA
SNAIL2	Forward: TGCCTGTCATACCACAACCAGA
	Reverse: GGAGGAGGTGTCAGATGGAGGA
ZEB1	Forward: ACCTGCCAACAGACCAGACAGT
	Reverse: ACATCCTGCTTCATCTGCCTGAG
FN1	Forward: CCGCCGAATGTAGGACAAGA
	Reverse: TGCCAACAGGATGACATGAAA
VIMENTIN	Forward: TGAACCTGAGGGAAACTAA
	Reverse: TCAAGGTCATCGTGATGCTG
CDH1	Forward: TCCCTTCACAGCAGAACTAACA
	Reverse: TCACCCACCTCTAAGGCCATC
SNAIL1	Forward: ACCACTATGCCGCGCTCTT
	Reverse: GGTCGTAGGGCTGCTGGAA
ZEB2	Forward: CCTACTAATTCAGCCATTACCCAGT
	Reverse: CCACTAAACCCGTGTGTAGCCAT
CDH2	Forward: ACTGCACAGATGTGGACAGG
	Reverse: GTTCTTTATCCCGGCGTTTC
CCND1	Forward: TTCCTGTCCTACTACCGCCTCA
	Reverse: CCTCAGATGTCCACGTCCC
CCNE1	Forward: GTGTGGGAGCCAGCCTTG
	Reverse: ATCATCTTCTTTGTCAGGTGTGG
KI-67	Forward: TGTGCAGAGAGTAACGCGGAGT
	Reverse: CTGTCCCTATGACTTCTGGTTC
IRE1α(ERN1)	Forward: GGGCGAACAGAATACACCATCACC
	Reverse: ACGTCCCCAGATTCACTGTCCAC
PERK(EIF2AK3)	Forward: TGTCATCCAGCCTTAGCAAACCAG
	Reverse: TCCATGCTTTCACGGTCTTGGTC
XBP1	Forward: CAGCTCAGACTGCCAGAGATCGAA
	Reverse: TCCGCCTCCTCTTCAGCAACCA
ATF4	Forward: GGTTCTCCAGCGACAAGG
	Reverse: TCTCCAACATCCAATCTGTCC
ATF6	Forward: AACTTTCCGTGACTAAACCTGT
	Reverse: CCTTTAATCTCGCCTCTAACCC

CRELD2	Forward: TGACGAGTCCTGCAAGACGTGCT
	Reverse: GCCGCACACTCGTCCACATCCAC
APMAP	Forward: CTGTCCTCCGAGACACCCAT
	Reverse: ACTTCCCTGGTCACAGTATCAT

Table S2

The siRNAs targeting PERK, IRE1α, ATF6, CREDL2, and APMAP.

Names	Sequence (5`-3`)
siPERK-sense	GUAGCUGGAAUGACAUAAATT
siPERK-antisense	UUUAUGUCAUUCCAGCUACTT
siIRE1α-sense	CCUUUCUCCCAGAUCCUAATT
siIRE1α-antisense	UUAGGAUCUGGGAGAAAGGTT
siATF6-sense	GUGAGCUACAAGUGUAUUATT
siATF6-antisense	UAAUACACUUGUAGCUCACTT
siCRELD2-sense	GCAGCGACUUCGAAUGCAATT
siCRELD2-antisense	UUGCAUUCGAAGUCGCUGCTT
siAPMAP-sense	UGUUCUGCAUCCAAAUACGTT
siAPMAP-antisense	CGUAUUUGGAUGCAGAACACC
siControl-sense	UUCUCCGAACGUGUCACGUTT
siControl-antisense	ACGUGACACGUUCGGAGAATT

Table S3

Primers used for the construction of overexpression plasmids.

Genes	Sequences (5`-3`)
ATF4	Forward: CGAAGATGGCAGCCCGACCCC
	Reverse: GCTCTAGACTAGGGGACCTTTTTCTTCC
APMAP	Forward: TTGAATTCATGAGCGAGGCGGACGGGC
	Reverse: ATTCTAGACTAAACAGCCTGGAGGCTGAGTCT

Table 4

Primers for the construction of luciferase reporters.

Gene	Sequence (5`-3`)
CRELD2（-1472 bp/+38 bp）	Forward:AAGGTACCCACTGTCCTGCACTAGCCCACT
	Reverse: TTAGATCTCTGTCCCCCAGGCTACTTGACG
CRELD2（-874 bp/+38 bp)	Forward:

	AAGGTACCGAGTTCTCTGAAATCCAGACGC<br>Reverse:<br>TTAGATCTCTGTCCCCCAGGCTACTTGACG

Supplementary Figures

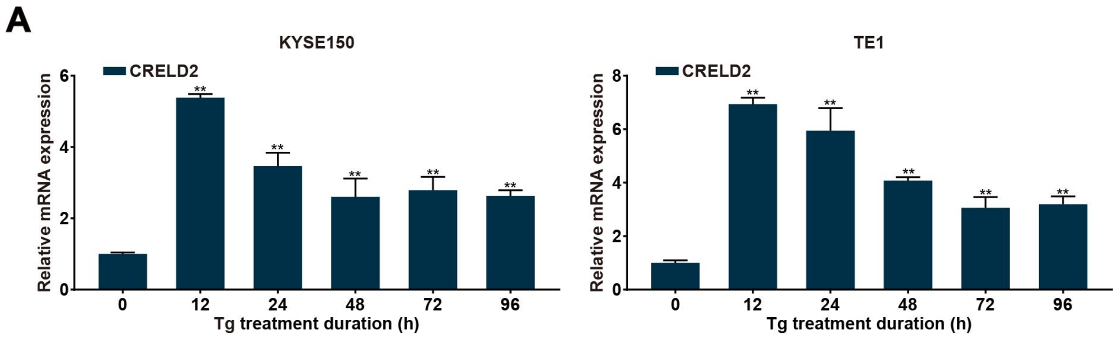

Supplementary Fig S1. Endoplasmic reticulum stress-induced alterations in CRELD2 expression. (A) qRT-PCR analysis of CRELD2 expression levels in KYSE150 and TE1 cells treated with 100nM thapsigargin (Tg) for specified treatment durations. Data represent the mean ± SD of three independent experiments. *P < 0.05, **P < 0.01.

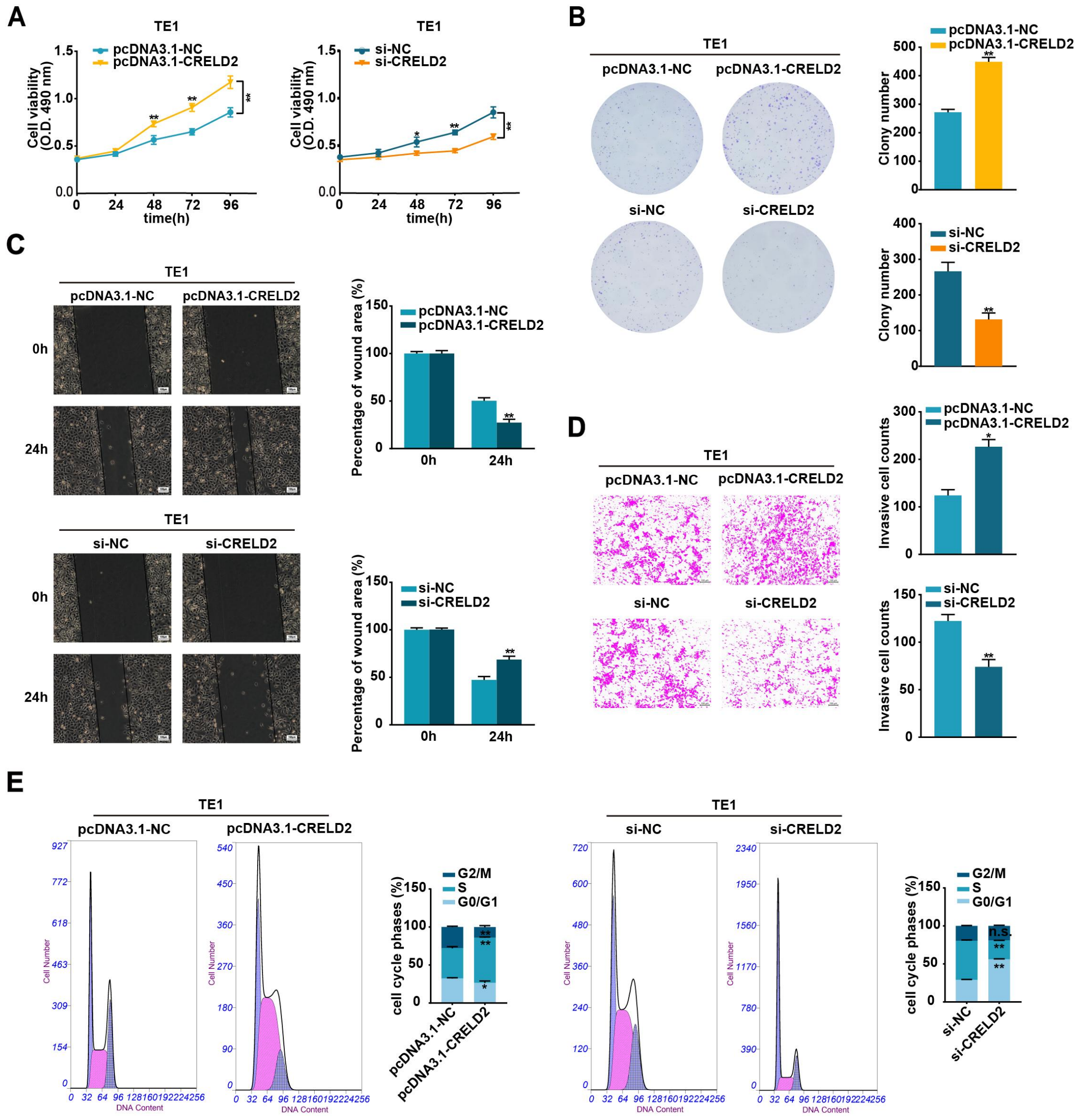

Supplementary Fig S2. CRELD2 promotes proliferation, migration, and invasion of TE1 cells. (A-B) The proliferation ability of CRELD2-overexpressing and CRELD2-knockdown TE1 cells was examined by MTS (A) and colony formation (B) assays. (C-D) Cell migration and invasion abilities were verified by wound healing (C) and transwell invasion (D) assays in CRELD2-overexpressing and CRELD2-knockdown cells. Scale bar, 100 μm. (F) Flow cytometric cell cycle analysis of TE1 cells with CRELD2 overexpression or knockdown. Data represent the

mean ± SD of three independent experiments. *P < 0.05, **P < 0.01, n.s., not significant.

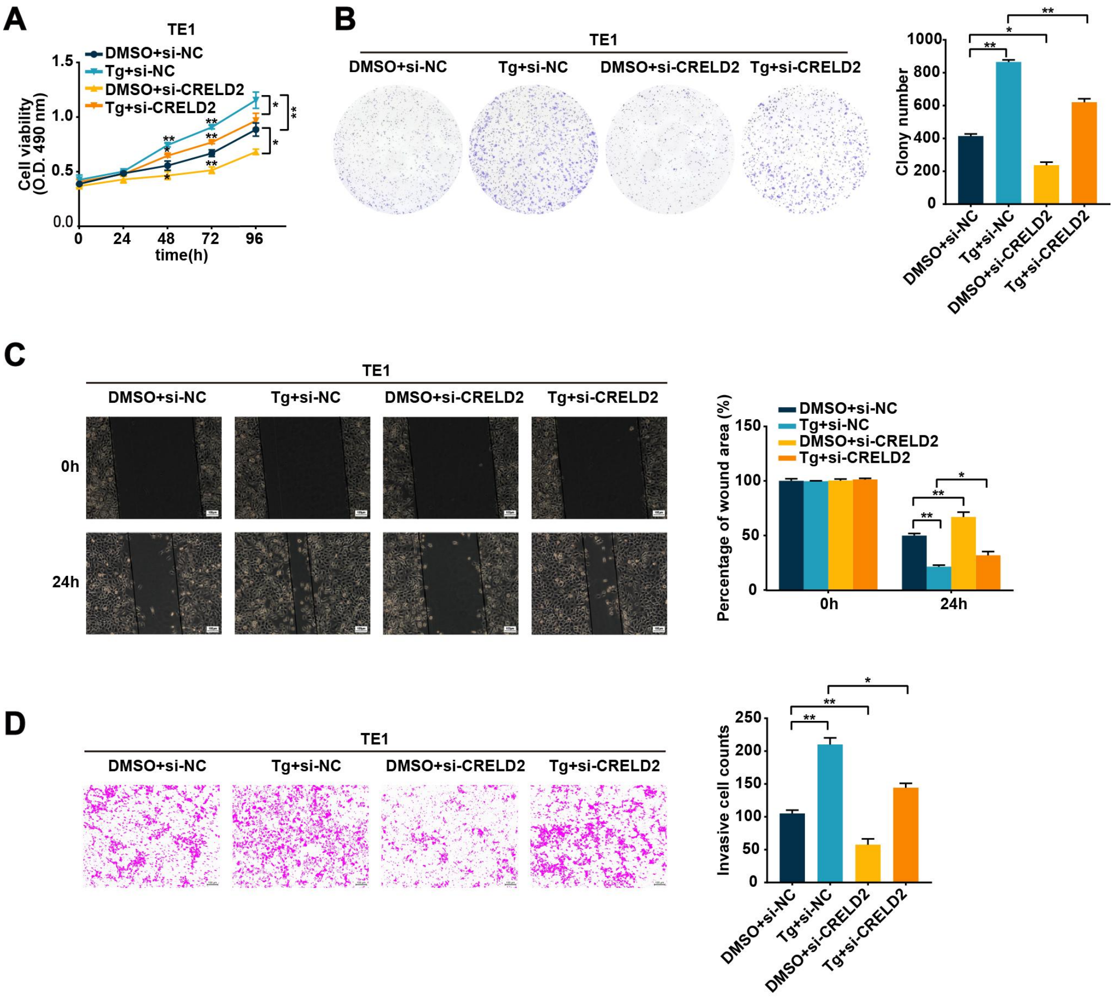

Supplementary Fig S3. CRELD2 mediates the ER stress-regulated malignant biological behaviour in TE1 cells. (A-B) The proliferation ability of the indicated cells was tested by MTS (A) and colony formation (B) assays. (C) Wound healing assay was conducted to explore the migration ability in indicated cells. Scale bar, 100 μm. (D) Transwell assay was performed to evaluate the invasion ability of the indicated cells. Scale bar, 100 μm. Data represent the mean ± SD of three independent experiments. A representative data from three independent experiments is shown. *P < 0.05, **P < 0.01.

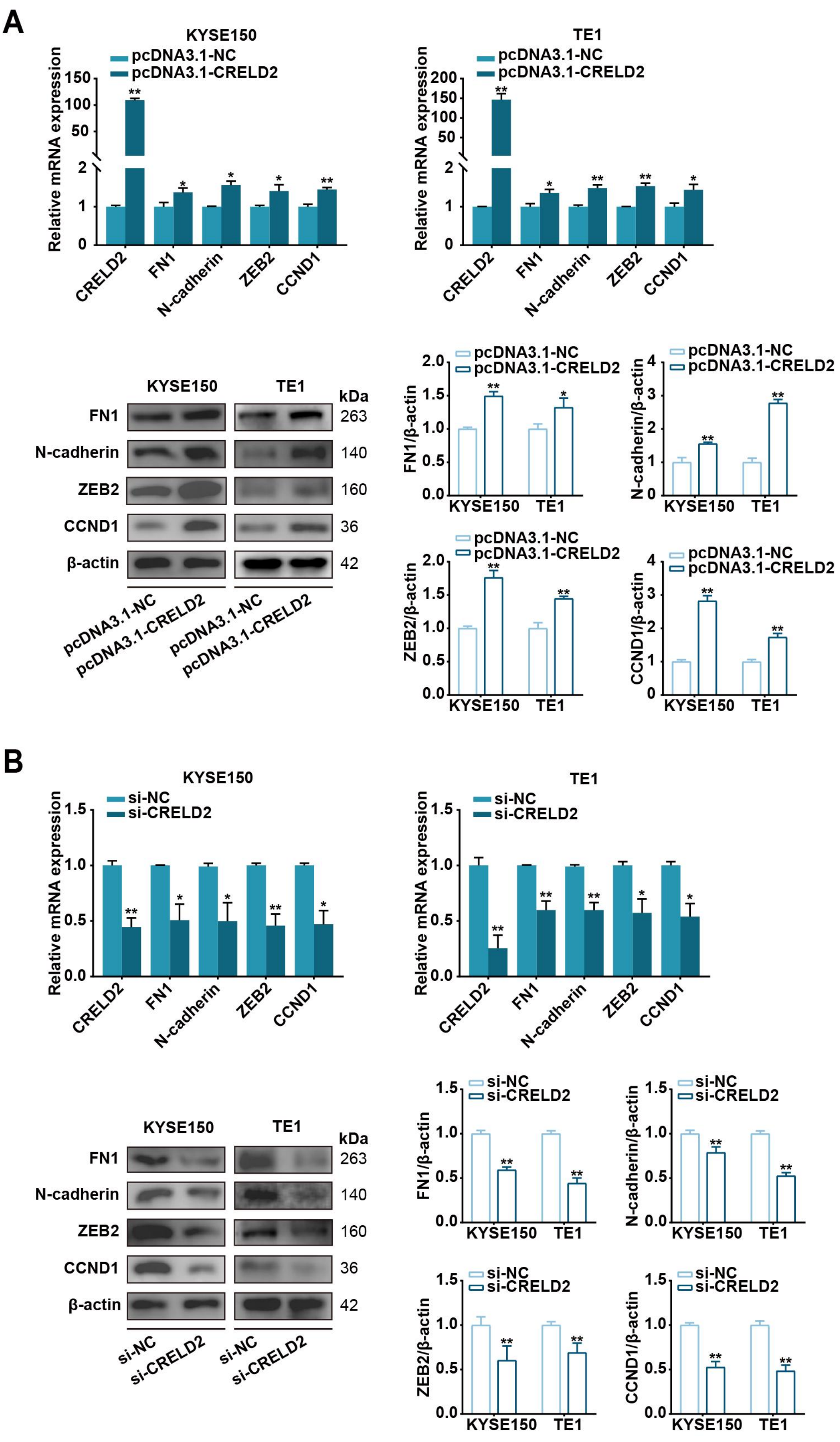

Supplementary Fig S4. Effects of CRELD2 on EMT and proliferation-related proteins. (A) qRT-PCR and Western blot analysis of EMT markers (FN1, N-cadherin,

and ZEB2) and proliferation-related factors (CCND1) in CRELD2-overexpression KYSE150 and TE1 cells. (B) qRT-PCR and Western blot analysis of EMT markers (FN1, N-cadherin, and ZEB2) and proliferation-related factors (CCND1) in CRELD2-knockdown KYSE150 and TE1 cells. The protein levels were quantified by band densitometry. Data represent the mean ± SD of three independent experiments. *P < 0.05, **P < 0.01.

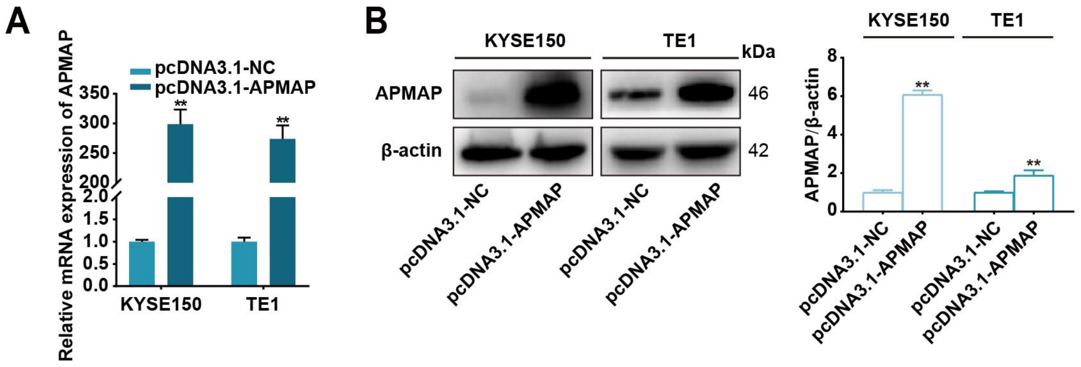

Supplementary Fig S5. Transfection efficiency of APMAP overexpression in KYSE150 and TE1 cells. (A-B) qRT-PCR (A) and Western blot (B) analysis of APMAP expression in KYSE150 and TE1 cells transfected with pcDNA3.1-NC or pcDNA3.1-APMAP. The protein levels were quantified by band densitometry. Data represent the mean ± SD of three independent experiments. *P < 0.05, **P < 0.01.
