# Supplementary material for: Endoplasmic reticulum stress-induced CRELD2 promotes APMAP-mediated activation of TGF-β/SMAD and NF-κB pathways in esophageal squamous cell carcinoma
Source: Front Immunol. 2025 Jul 31;16:1616201. doi: 10.3389/fimmu.2025.1616201 (PMC12351132; doi:10.3389/fimmu.2025.1616201)
Supplement: Supplementary file 2 [file DataSheet2.pdf]

**A**

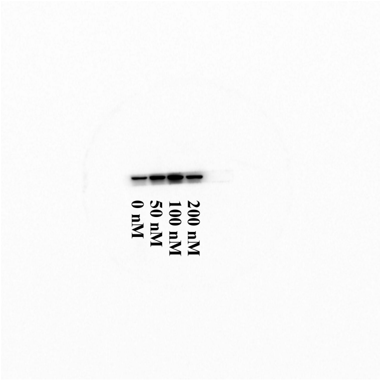


XBP1s (protein bands of XBP1s in Tg (0, 50, 100, and 200 nM) treated TE1 cells in Fig. 1A).

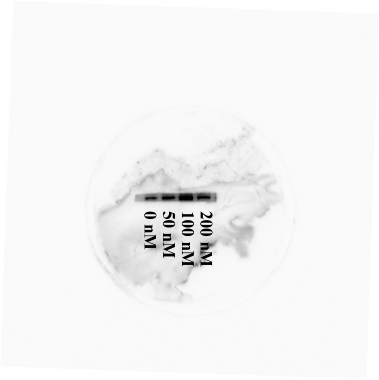


ATF4 (protein bands of ATF4 in Tg (0, 50, 100, and 200 nM) treated TE1 cells in Fig. 1A).

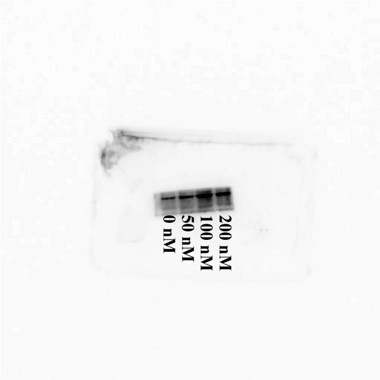


ATF6 (protein bands of ATF6 in Tg (0, 50, 100, and 200 nM) treated TE1 cells in Fig. 1A).

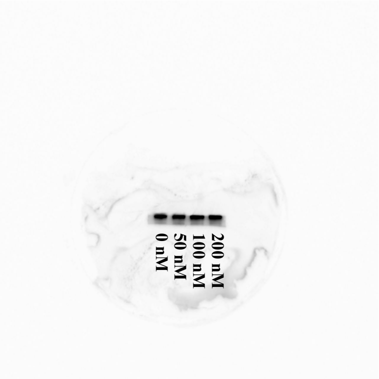


β-actin (protein bands of β-actin in Tg (0, 50, 100, and 200 nM) treated TE1 cells in Fig. 1A).

**B**

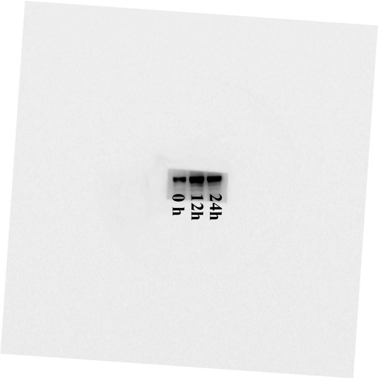


XBP1s (protein bands of XBP1s in TE1 cells treated with 100 nM Tg at different time points (0, 12, and 24h) in Fig. 1B).

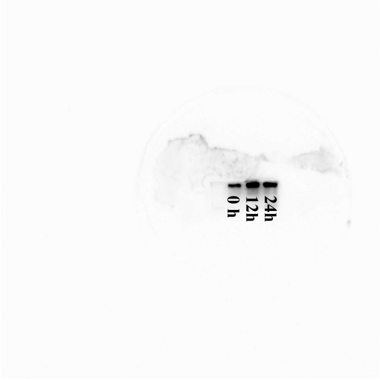


ATF4 (protein bands of ATF4 in TE1 cells treated with 100 nM Tg at different time points (0, 12, and 24h) in Fig. 1B).

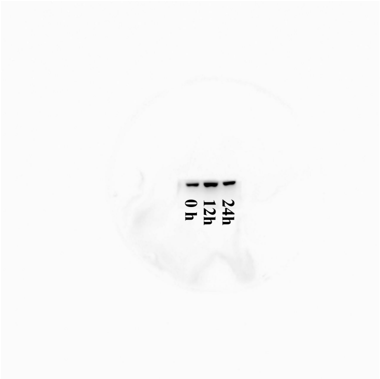


ATF6 (protein bands of ATF6 in TE1 cells treated with 100 nM Tg at different time points (0, 12, and 24h) in Fig. 1B).

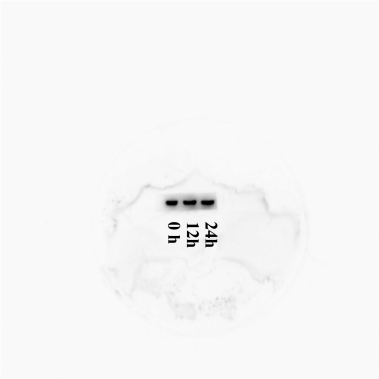


β-actin (protein bands of β-actin in TE1 cells treated with 100 nM Tg at different time points (0, 12, and 24h) in Fig. 1B).

**C**

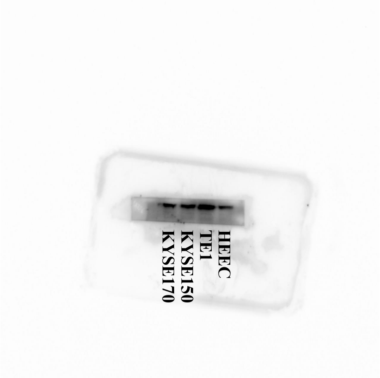


CRELD2 (protein bands of CRELD2 in human normal esophageal epithelial cells (HEEC) and ESCC cell lines (TE1, KYSE150, and KYSE170) in Fig.1E)

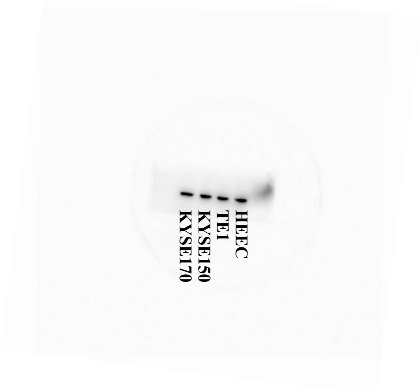


β-actin (protein bands of β-actin in HEEC and ESCC cell lines (TE1, KYSE150, and KYSE170) in Fig.1E)

D

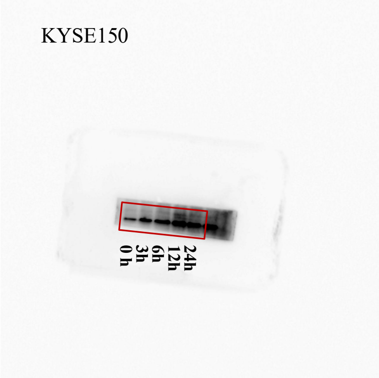


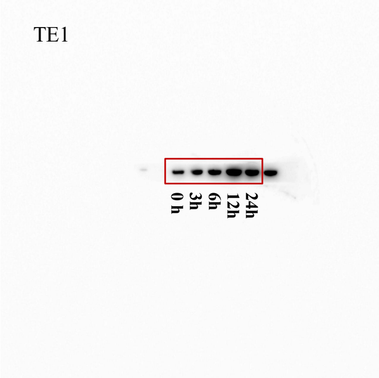


CRELD2 (protein bands of CRELD2 in KYSE150 and TE1 cells treated with 100 nM Tg at different time points (0, 3, 6, 12, and 24h) in Fig. 1F and Fig. 1G).

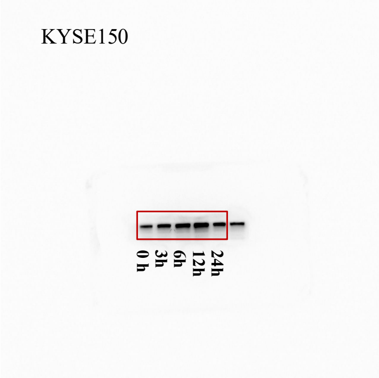


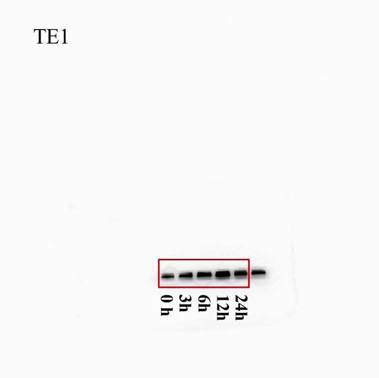


XBP1s (protein bands of XBP1s in KYSE150 and TE1 cells treated with 100 nM Tg at different time points (0, 3, 6, 12, and 24h) in Fig. 1F and Fig. 1G).

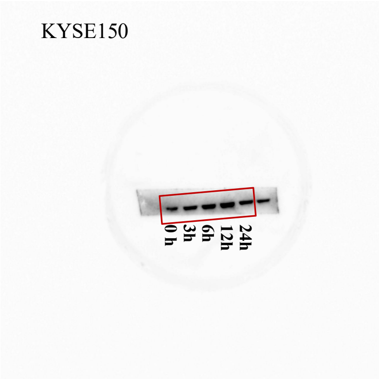


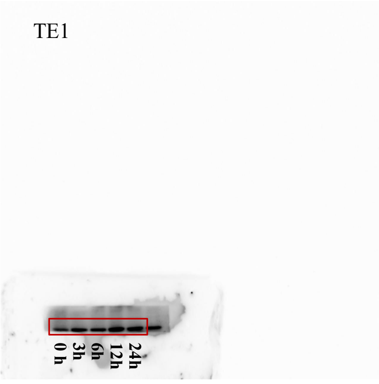


ATF4 (protein bands of ATF4 in KYSE150 and TE1 cells treated with 100 nM Tg at different time points (0, 3, 6, 12, and 24h) in Fig. 1F and Fig. 1G).

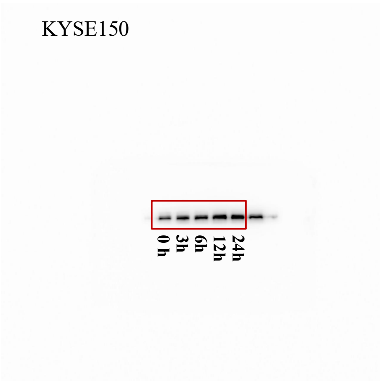


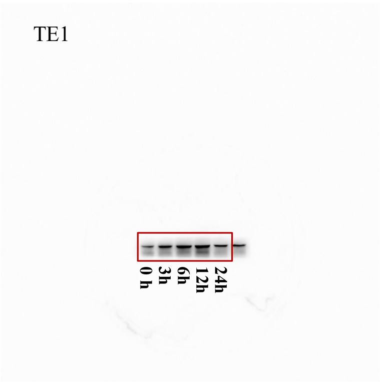


ATF6 (protein bands of ATF6 in KYSE150 and TE1 cells treated with 100 nM Tg at different time points (0, 3, 6, 12, and 24h) in Fig. 1F and Fig. 1G).

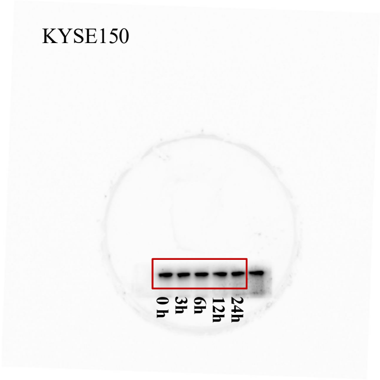


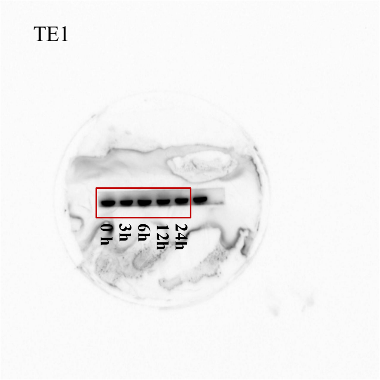


β-actin (protein bands of β-actin in KYSE150 and TE1 cells treated with 100 nM Tg at different time points (0, 3, 6, 12, and 24h) in Fig. 1F and Fig. 1G).

**E**

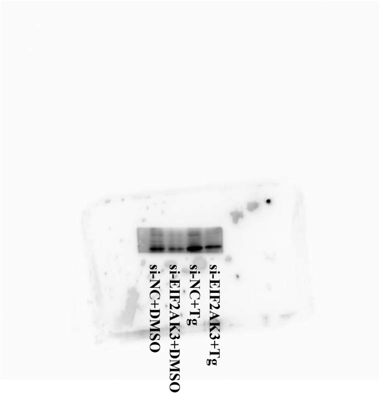


CRELD2 (protein bands of CRELD2 in siRNA-transfected KYSE150 cells treated with 100 nM Tg or DMSO for 12 h in Fig. 2C).

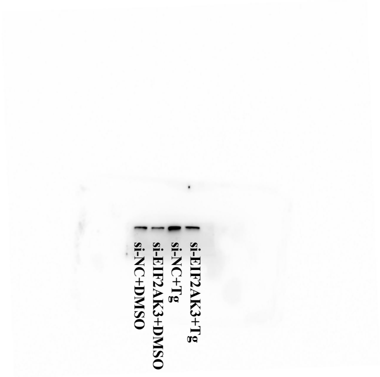


ATF4 (protein bands of ATF4 in siRNA-transfected KYSE150 cells treated with 100 nM Tg or DMSO for 12 h in Fig. 2C).

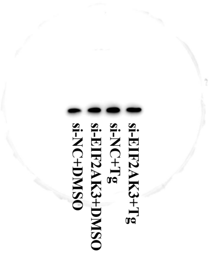


β-actin (protein bands of β-actin in siRNA-transfected KYSE150 cells treated with 100 nM Tg or DMSO for 12 h in Fig. 2C).

**F**

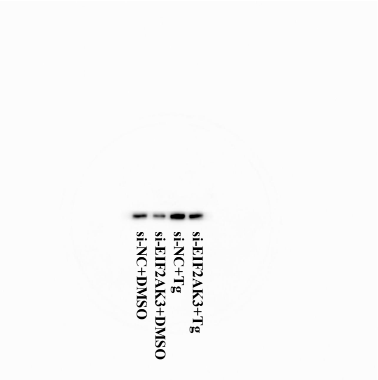


CRELD2 (protein bands of CRELD2 in siRNA-transfected TE1 cells treated with 100 nM Tg or DMSO for 12 h in Fig. 2D).

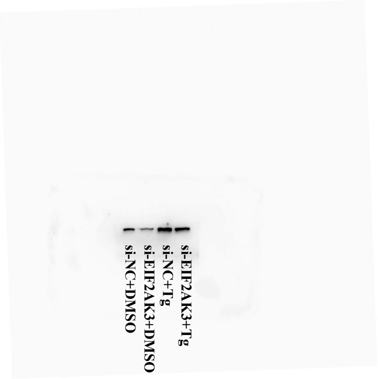


ATF4 (protein bands of ATF4 in siRNA-transfected TE1 cells treated with 100 nM Tg or DMSO for 12 h in Fig. 2D).

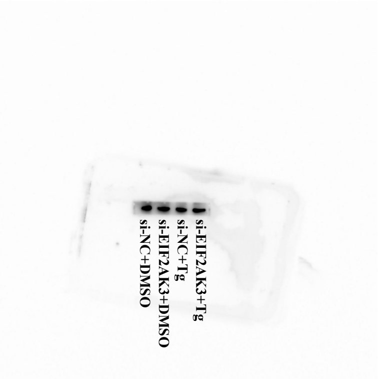


β-actin (protein bands of β-actin in siRNA-transfected TE1 cells treated with 100 nM Tg or DMSO for 12 h in Fig. 2D).

**G**

KYSE150

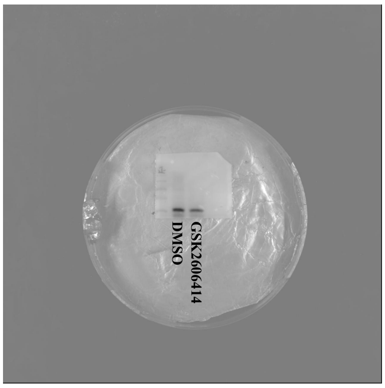


TE1

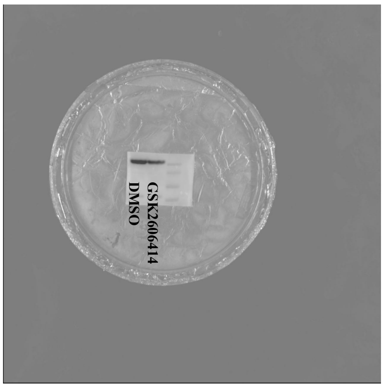


ATF4 (protein bands of ATF4 in KYSE150 and TE1 cells treated with DMSO or GSK2606414 in Fig. 2F).

KYSE150

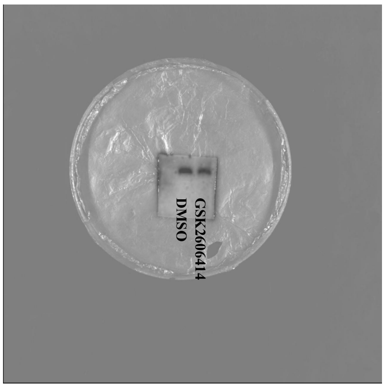


TE1

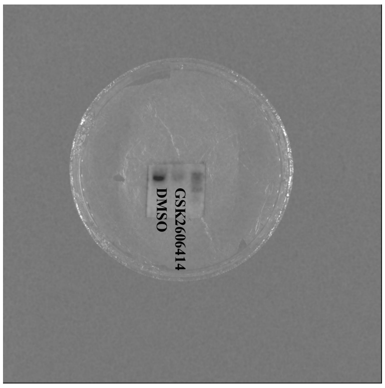


CRELD2 (protein bands of CRELD2 in KYSE150 and TE1 cells treated with DMSO or GSK2606414 in Fig. 2F).

KYSE150

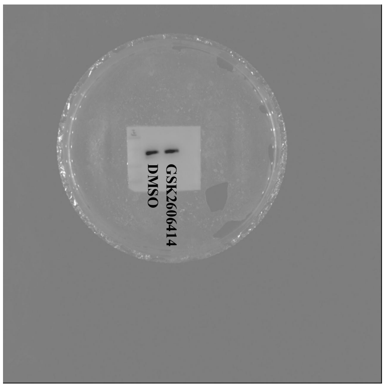


TE1

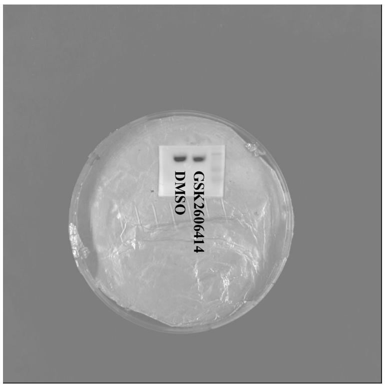


β-actin (protein bands of β-actin in KYSE150 and TE1 cells treated with DMSO or GSK2606414 in Fig. 2F.

**H**

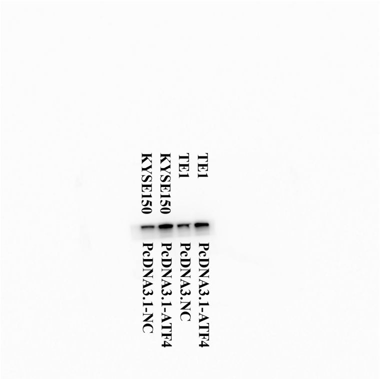


ATF4 (protein bands of ATF4 in ATF4-overexpressing KYSE150 and TE1 cells in Fig. 2H).

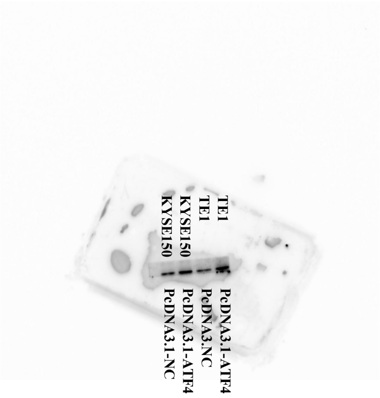


CRELD2 (protein bands of CRELD2 in ATF4-overexpressing KYSE150 and TE1 cells in Fig. 2H).

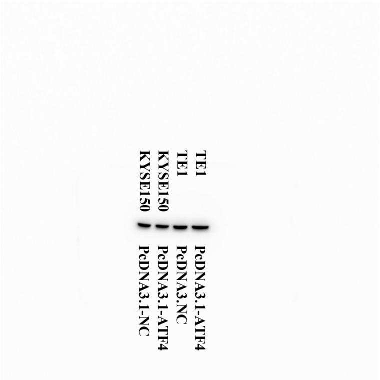


β-actin (protein bands of β-actin in ATF4-overexpressing KYSE150 and TE1 cells in Fig. 2H).

**I**

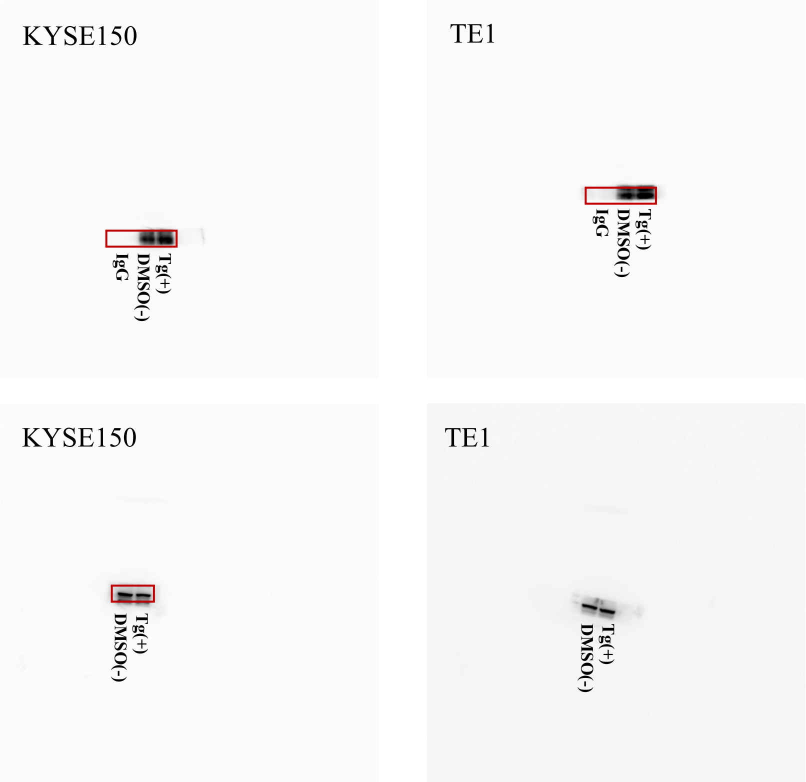


APMAP (protein bands of APMAP in KYSE150 and TE1 cells treated with Tg (+) or DMSO (-) in Fig. 5A).

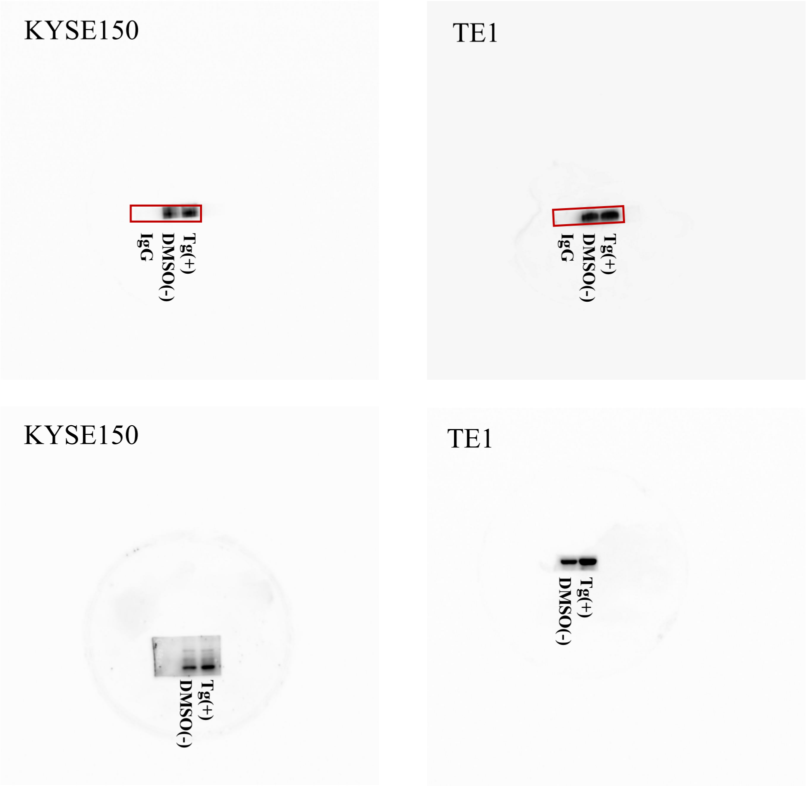


CRELD2 (protein bands of CRELD2 in KYSE150 and TE1 cells treated with Tg (+) or DMSO (-) in Fig. 5A).

**J**

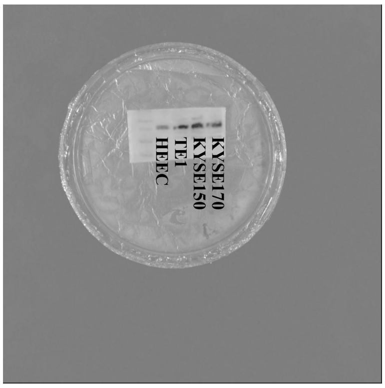


APMAP (protein bands of APMAP in HEEC and ESCC cell lines (TE1, KYSE150, and KYSE170) in Fig. 5D).

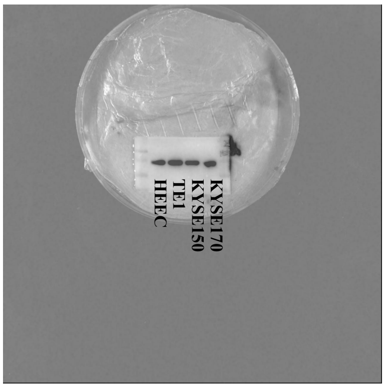


β-actin (protein bands of β-actin in HEEC and ESCC cell lines (TE1, KYSE150, and KYSE170) in Fig. 5D).

**K**

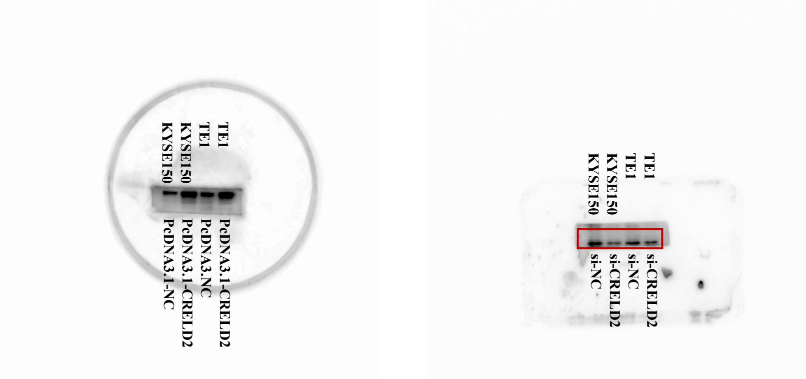


CRELD2 (protein bands of CRELD2 in CRELD2-overexpressing and CRELD2-knockdown KYSE150 and TE1 cells in Fig. 5E).

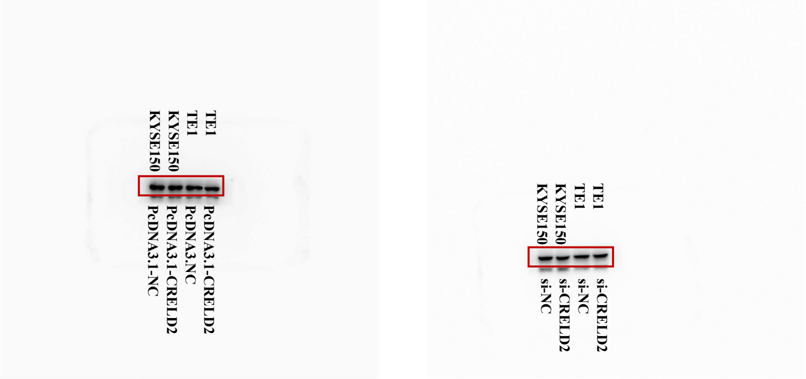


APMAP (protein bands of APMAP in CRELD2-overexpressing and CRELD2-knockdown KYSE150 and TE1 cells in Fig. 5E).

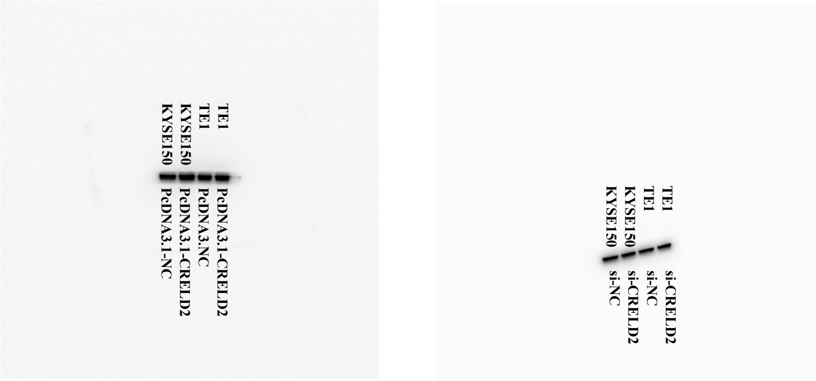


β-actin (protein bands of β-actin in CRELD2-overexpressing and CRELD2-knockdown KYSE150 and TE1 cells in Fig. 5E).

**L**

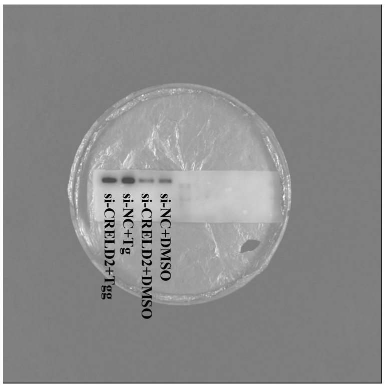


APMAP (protein bands of APMAP in the membrane fraction of KYSE150 cells in Fig. 5H).

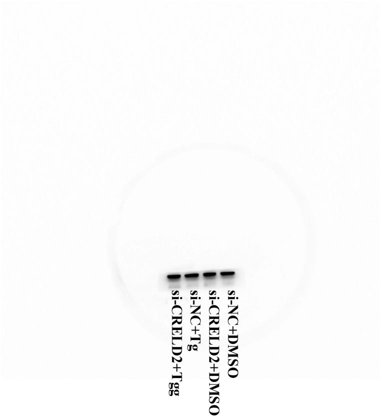


ATP1A1 (protein bands of ATP1A1 in the membrane fraction of KYSE150 cells in Fig. 5H).

M

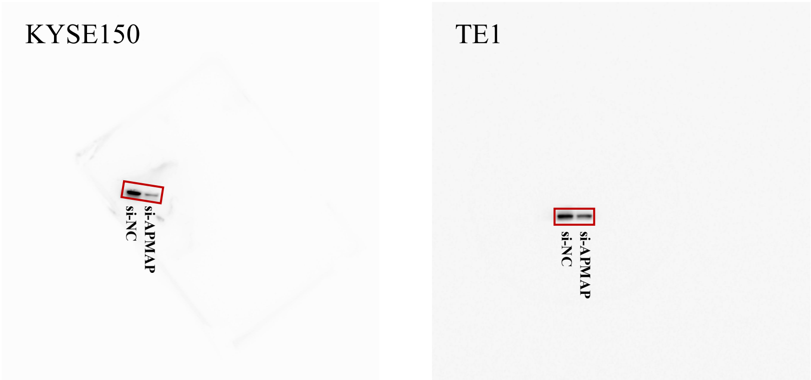


FN1 (protein bands of FN1 in siAPMAP-transfected cells in Fig. 7B).

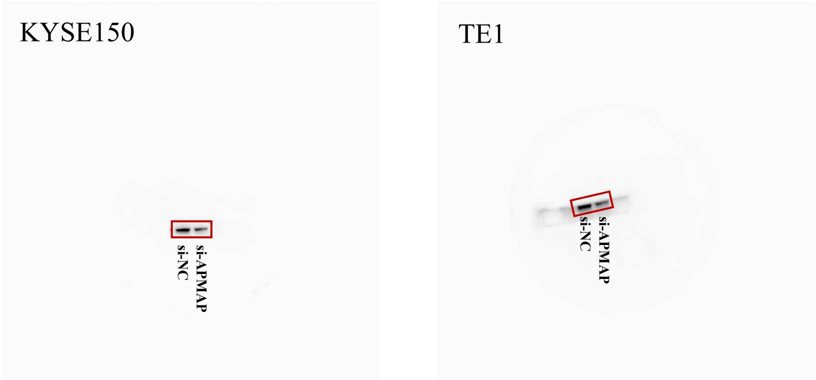


N-cadherin (protein bands of N-cadherin in siAPMAP-transfected cells in Fig. 7B).

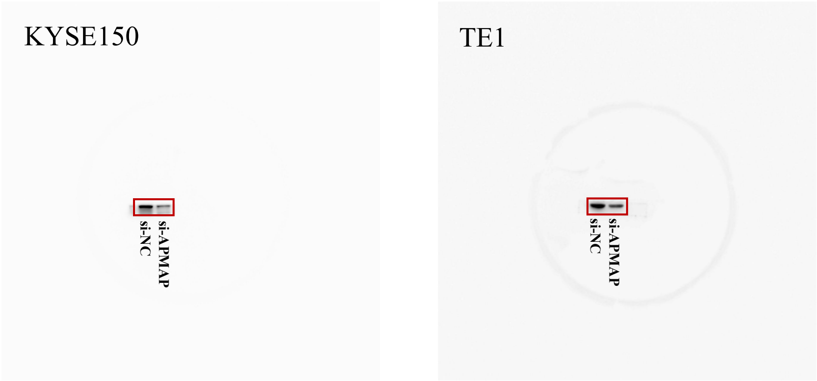


ZEB2 (protein bands of ZEB2 in siAPMAP-transfected cells in Fig. 7B).

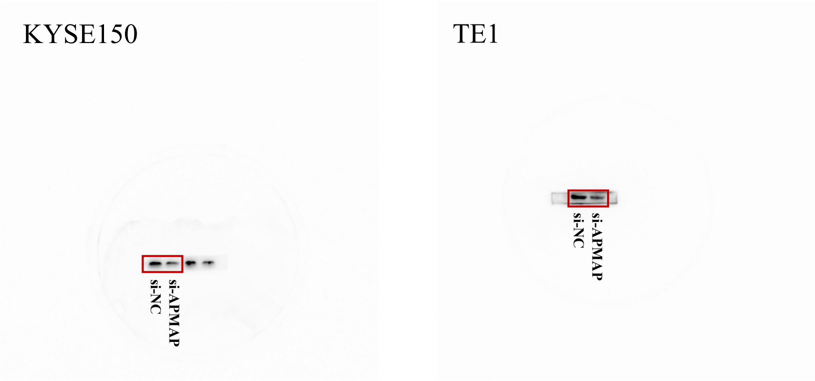


CCND1 (protein bands of CCND1 in siAPMAP-transfected cells in Fig. 7B).

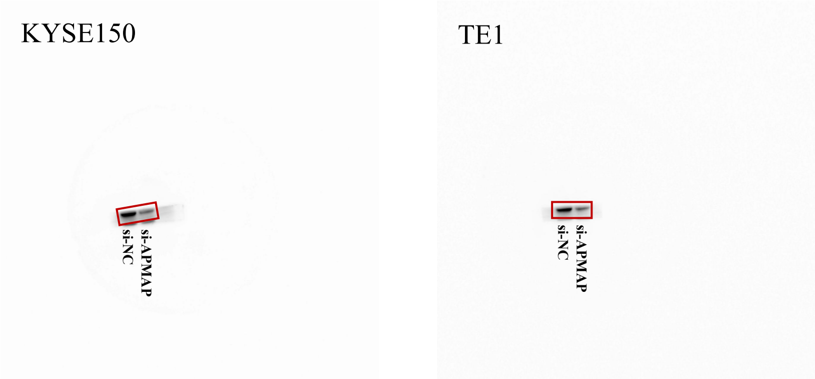


APMAP (protein bands of APMAP in siAPMAP-transfected cells in Fig. 7B).

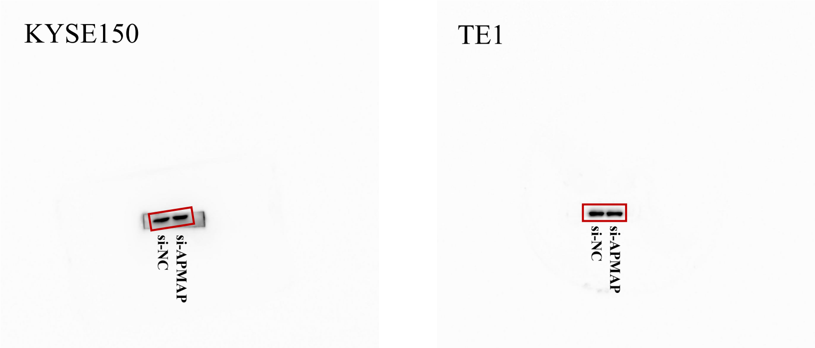


β-actin (protein bands of β-actin in siAPMAP-transfected cells in Fig. 7B).

**N**

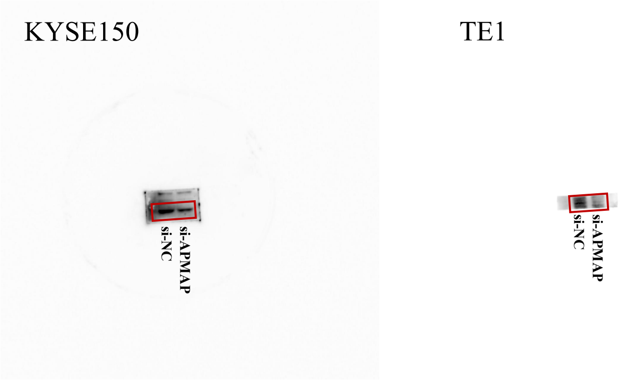


p-SMAD2 (protein bands of p-SMAD2 in siAPMAP-transfected KYSE150 and TE1 cells in Fig. 7C).

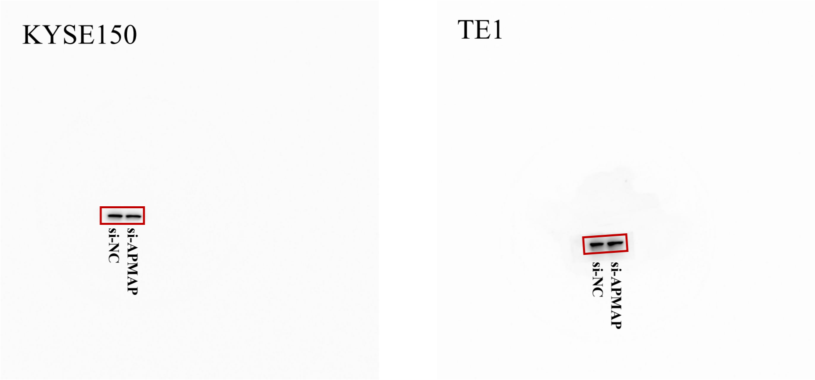


SMAD2 (protein bands of SMAD2 in siAPMAP-transfected KYSE150 and TE1 cells in Fig. 7C).

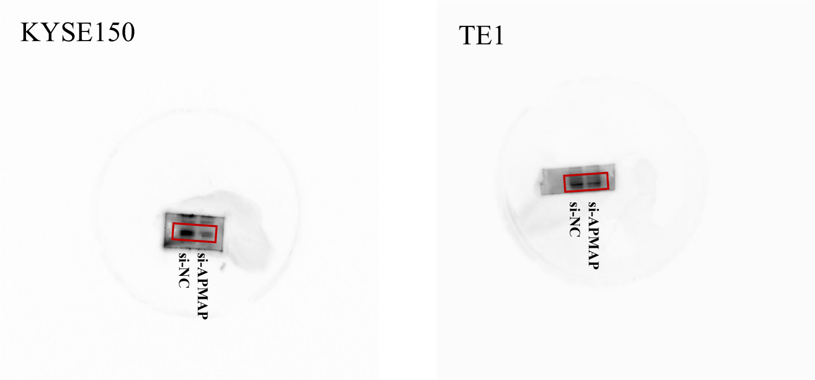


p-SMAD3 (protein bands of p-SMAD3 in siAPMAP-transfected KYSE150 and TE1 cells in Fig. 7C).

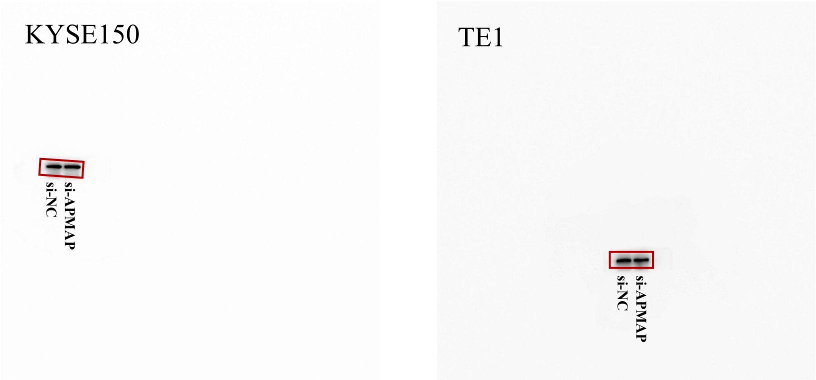


SMAD3 (protein bands of SMAD3 in siAPMAP-transfected KYSE150 and TE1 cells in Fig. 7C).

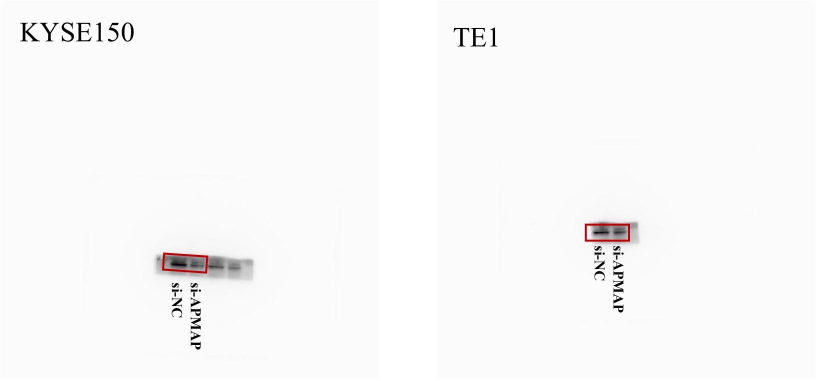


p-NFκB p65(protein bands of p-NFκB p65 in siAPMAP-transfected KYSE150 and TE1 cells in Fig. 7C).

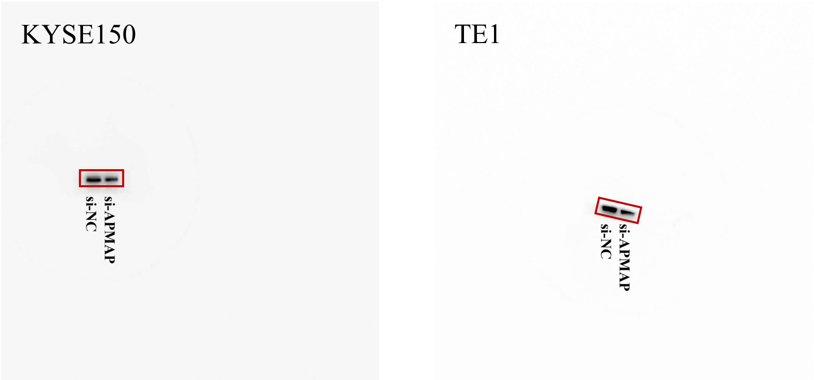


p-STAT3 (protein bands of p-STAT3 in siAPMAP-transfected KYSE150 and TE1 cells in Fig. 7C).

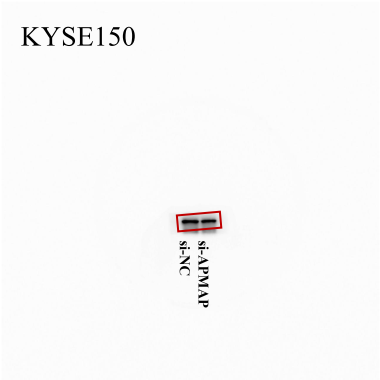


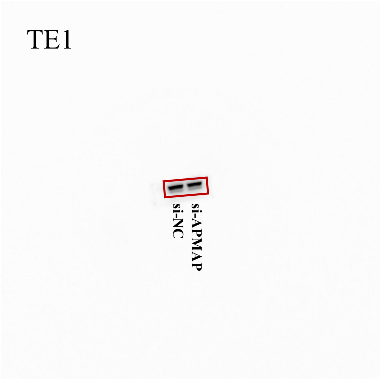


STAT3 (protein bands of STAT3 in siAPMAP-transfected KYSE150 and TE1 cells in Fig. 7C).

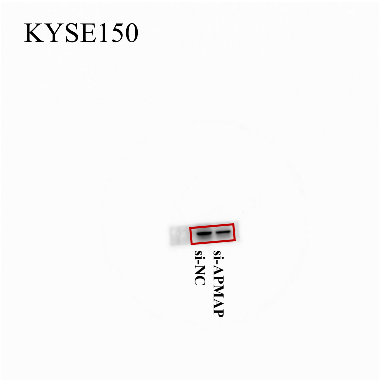


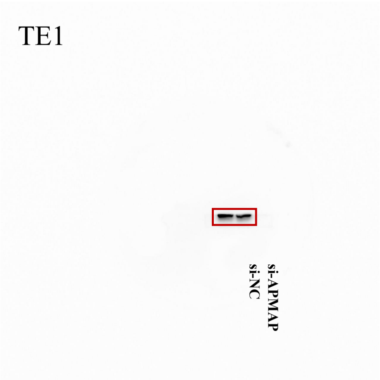


β-catenin (protein bands of β-catenin in siAPMAP-transfected KYSE150 and TE1 cells in Fig. 7C).

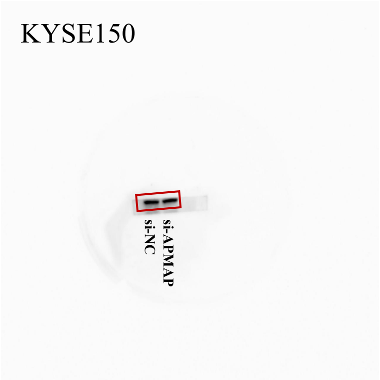


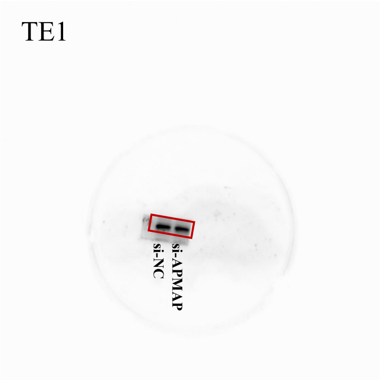


p-AKT (protein bands of p-AKT in siAPMAP-transfected KYSE150 and TE1 cells in Fig. 7C).

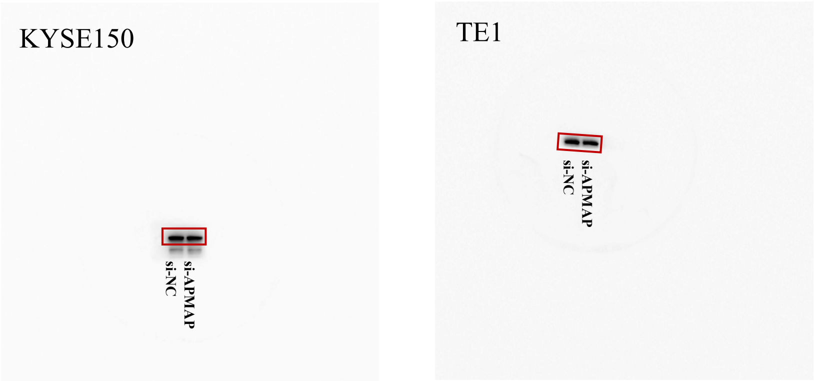


AKT (protein bands of AKT in siAPMAP-transfected KYSE150 and TE1 cells in Fig. 7C).

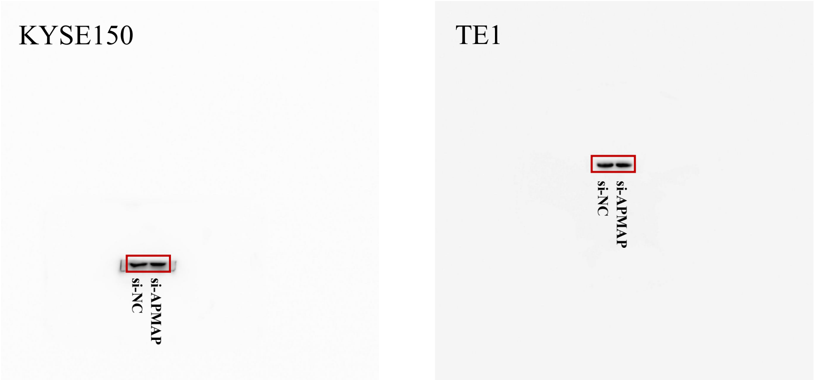


β-actin (protein bands of β-actin in siAPMAP-transfected KYSE150 and TE1 cells in Fig. 7C).

**O**

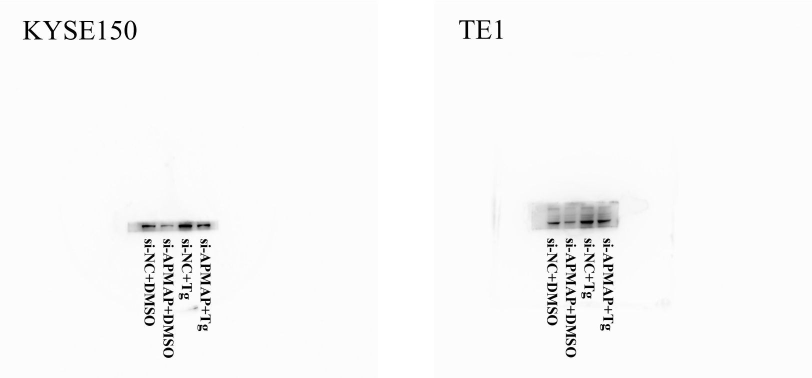


p-SMAD2 (protein bands of P-SMAD2 in indicated KYSE150 and TE1 cells in Fig. 7D).

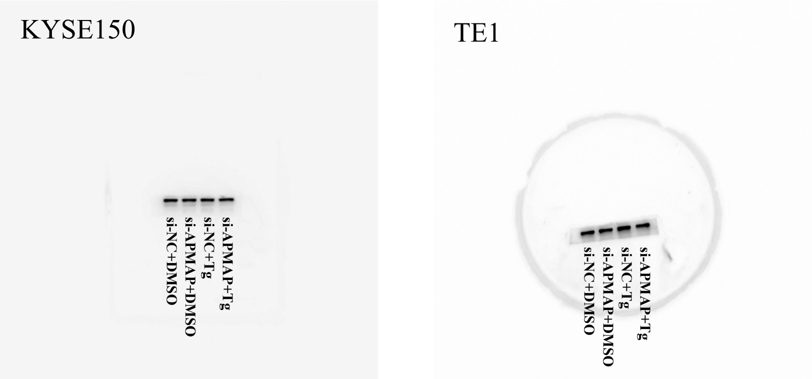


SMAD2 (protein bands of SMAD2 in indicated KYSE150 and TE1 cells in Fig. 7D).

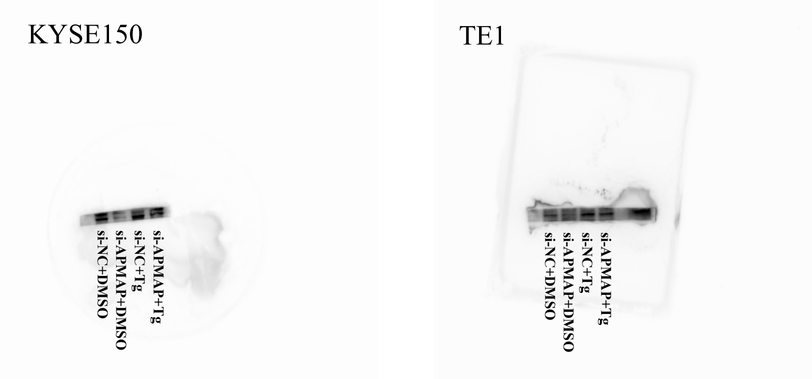


p-SMAD3 (protein bands of P-SMAD3 in indicated KYSE150 and TE1 cells in Fig. 7D).

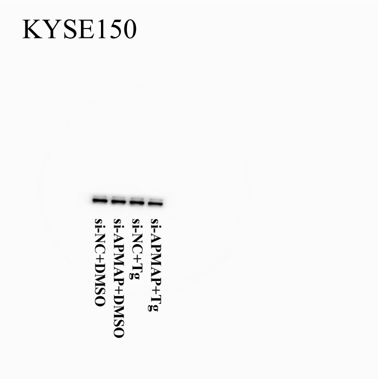

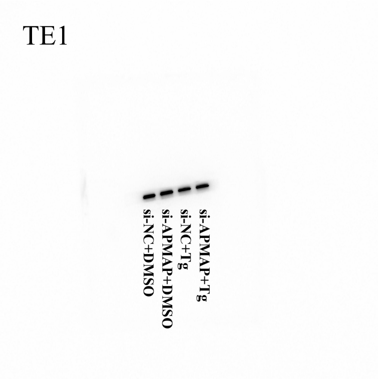


SMAD3 (protein bands of SMAD3 in indicated KYSE150 and TE1 cells in Fig. 7D).

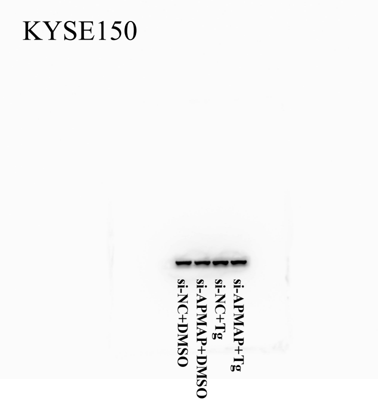

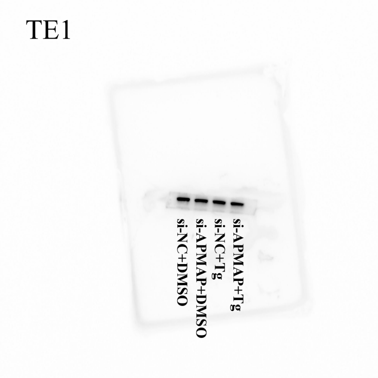


β-actin (protein bands of β-actin in indicated KYSE150 and TE1 cells in Fig. 7D).

**P**

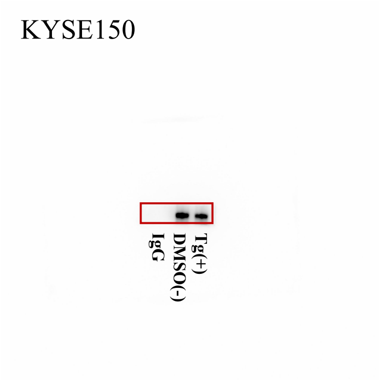


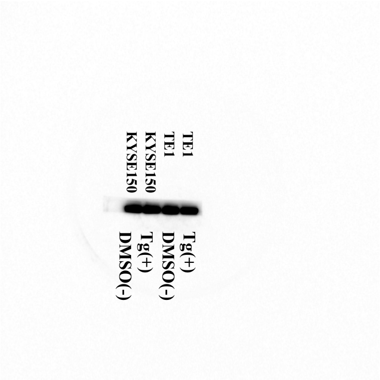


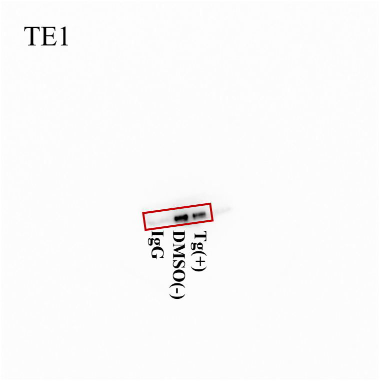


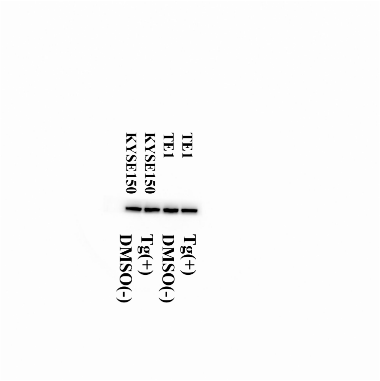


TAK1 (protein bands of TAK1 interacting with TGFBR1 in KYSE150 and TE1 cells in Fig. 7E).

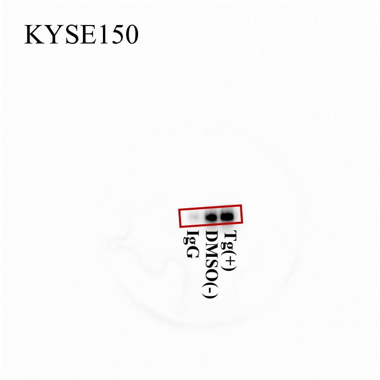


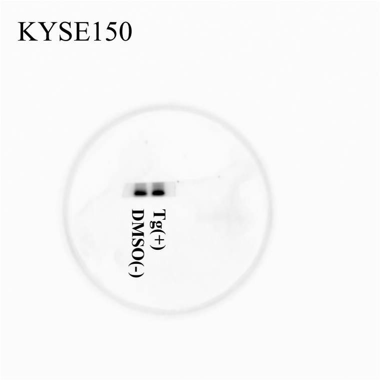

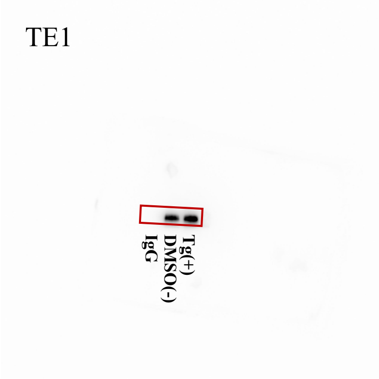


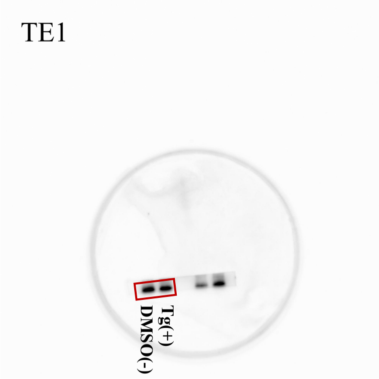


TGFBR1 (protein bands of TGFBR1 interacting with TGFBR1 in KYSE150 and TE1 cells in Fig. 7E).

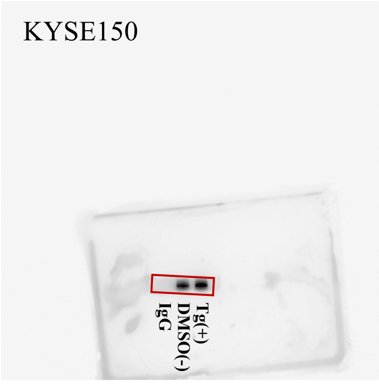


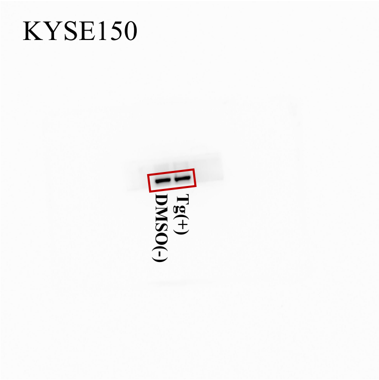


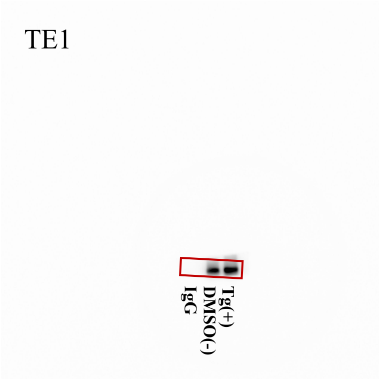


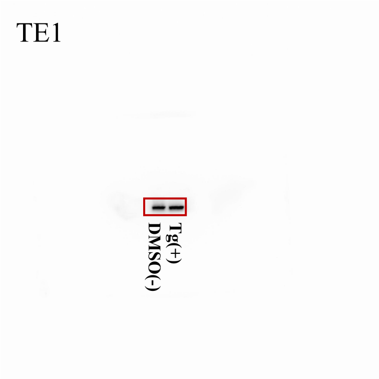


TAK1 (protein bands of TAK1 interacting with APMAP in KYSE150 and TE1 cells in Fig. 7E).

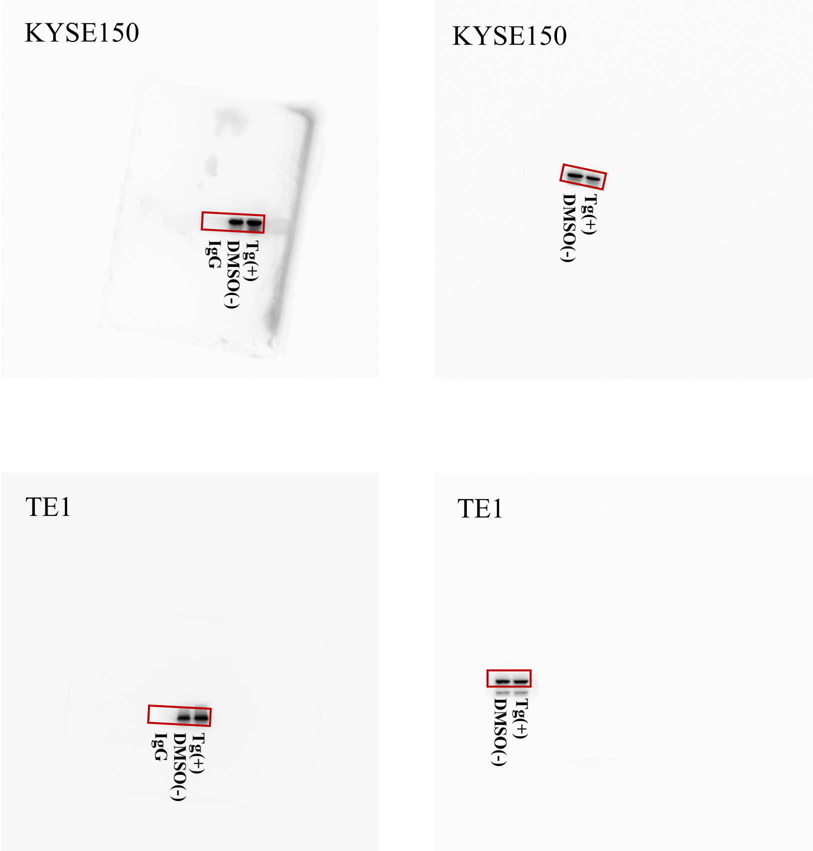


APMAP (protein bands of APMAP interacting with APMAP in KYSE150 and TE1 cells in Fig. 7E).

**Q**

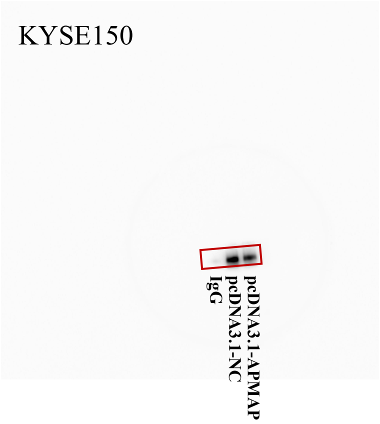


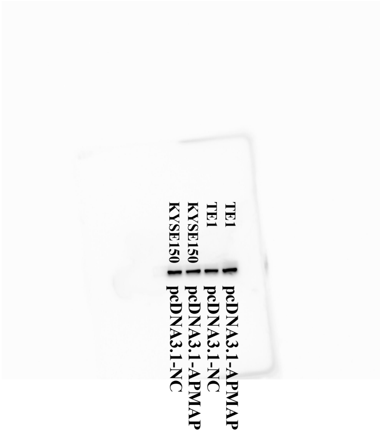


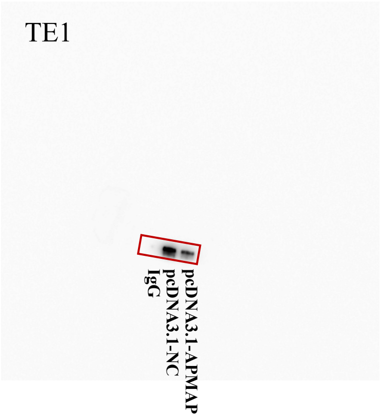


TAK1 (protein bands of TAK1 in APMAP-overexpressing KYSE150 and TE1 cells in Fig. 7F).

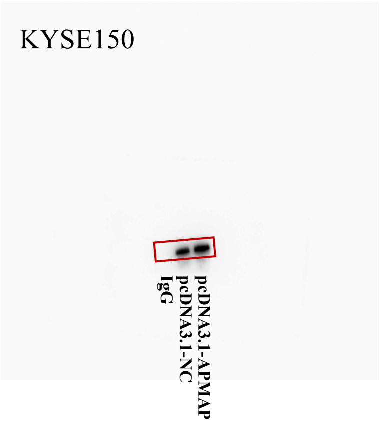


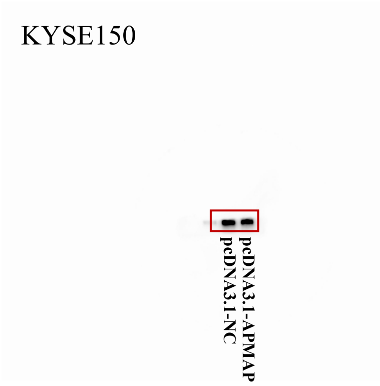

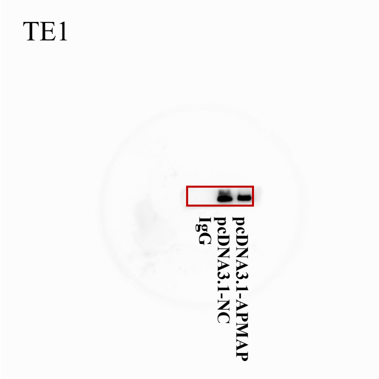


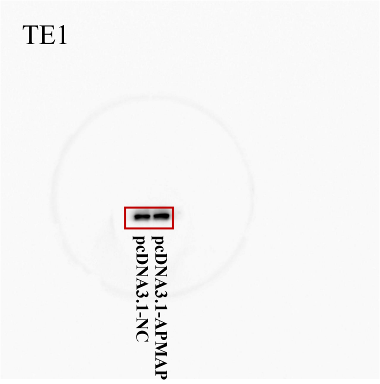


TGFBR1 (protein bands of TGFBR1 in APMAP-overexpressing KYSE150 and TE1 cells in Fig. 7F).

**R**

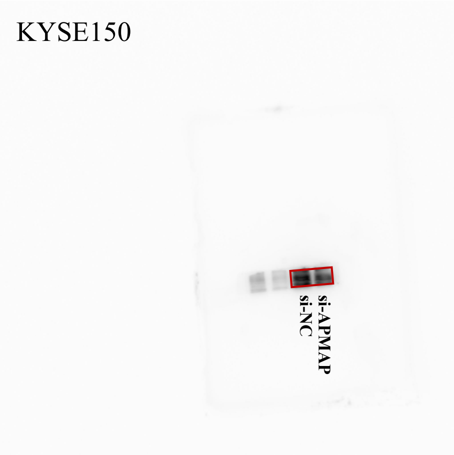


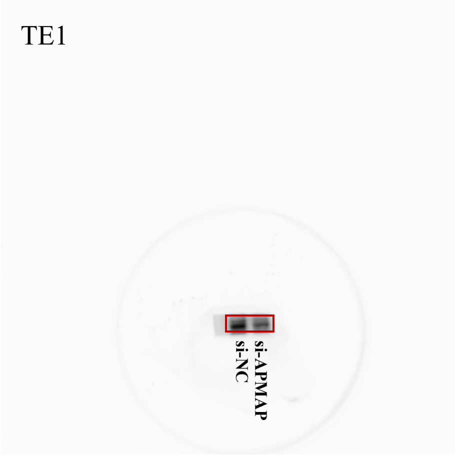


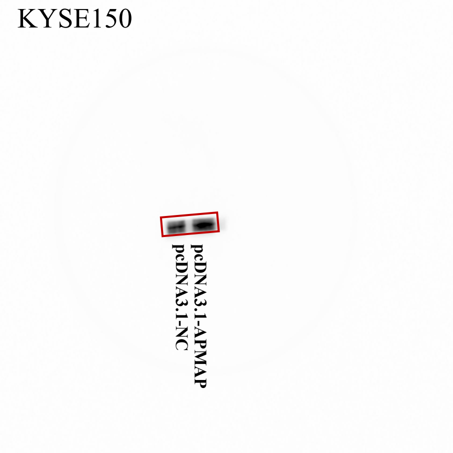


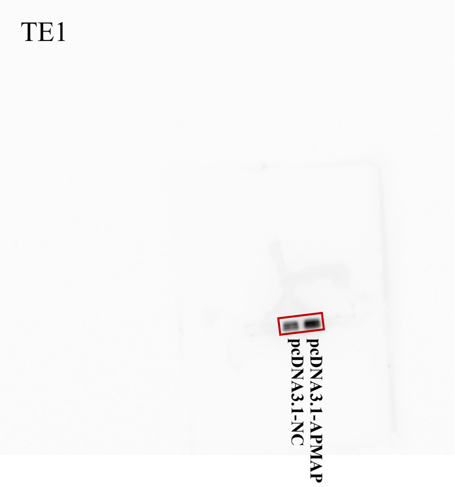


p-TAK1 (protein bands of p-TAK1 in KYSE150 and TE1 cells in Fig. 7G).

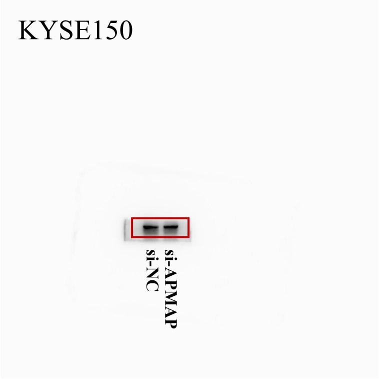


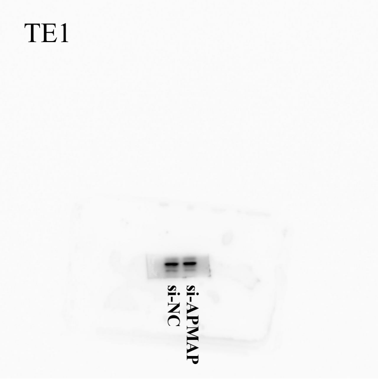

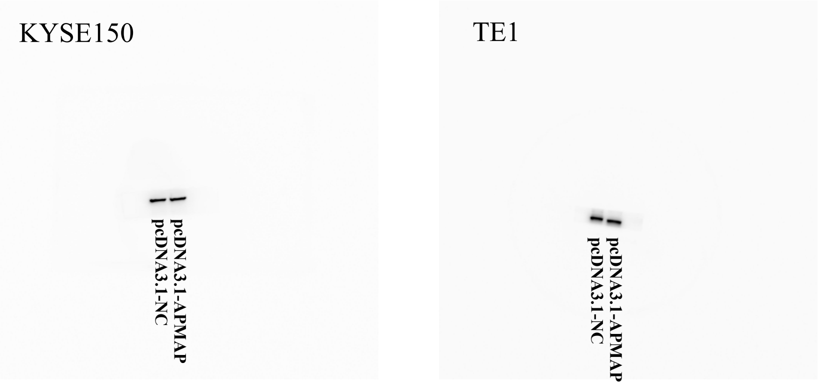


TAK1 (protein bands of TAK1 in KYSE150 and TE1 cells in Fig. 7G).

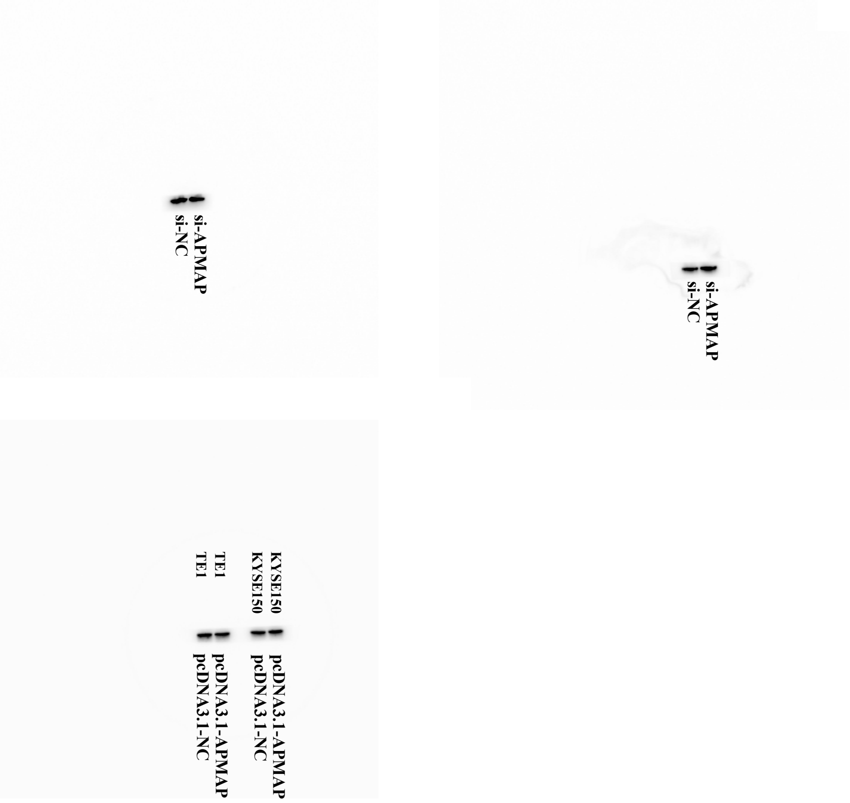


β-actin (protein bands of β-actin in KYSE150 and TE1 cells in Fig. 7G).

**S**

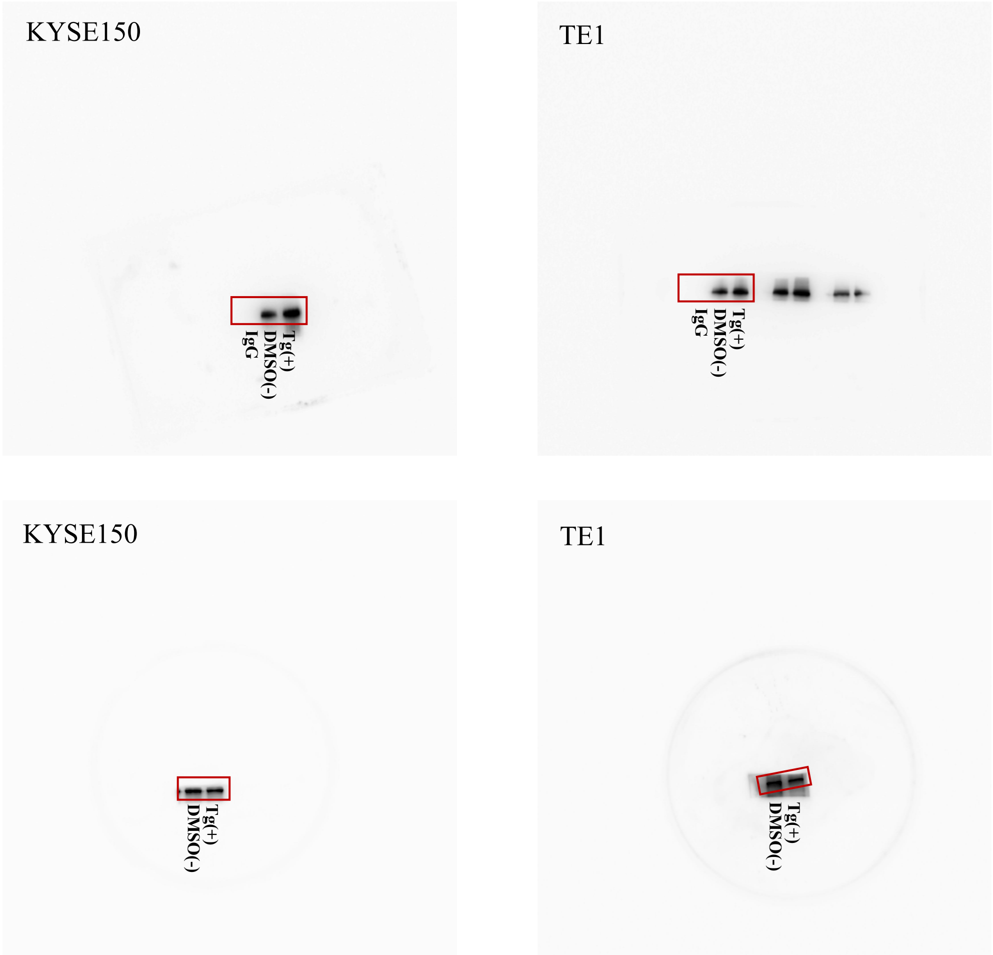


TAB1 (protein bands of TAB1 in KYSE150 and TE1 cells in Fig. 7H).

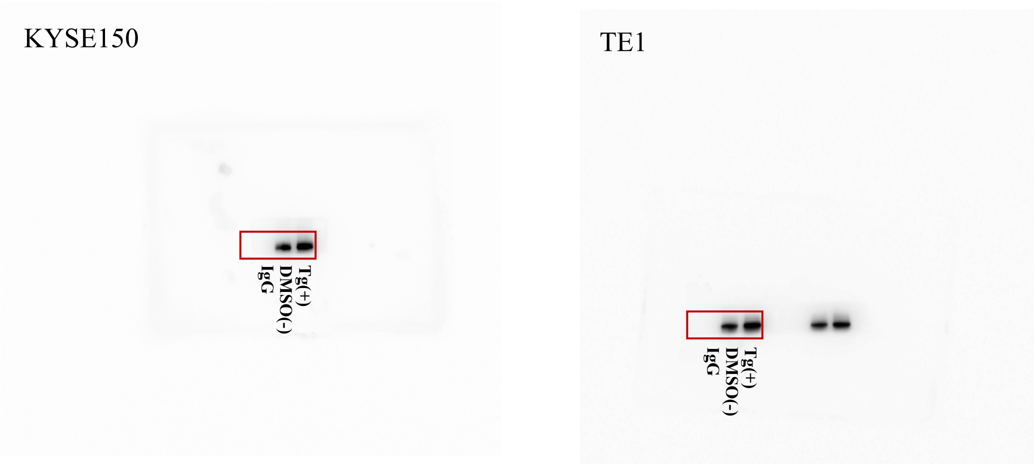

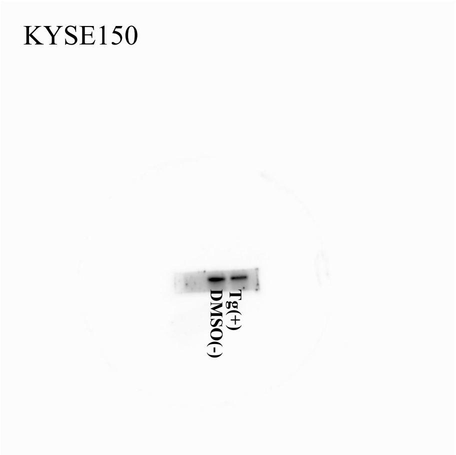


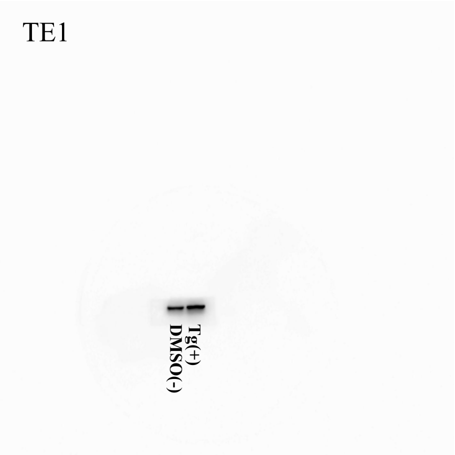


TAB2 (protein bands of TAB2 in KYSE150 and TE1 cells in Fig. 7H).

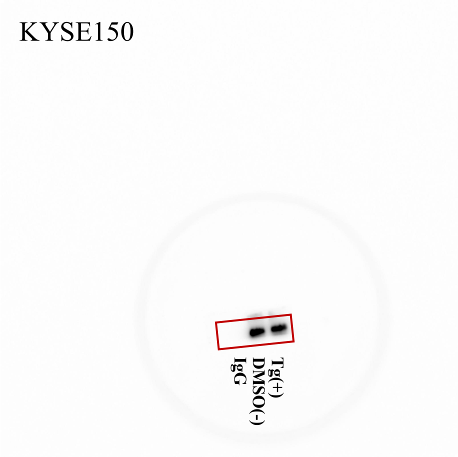


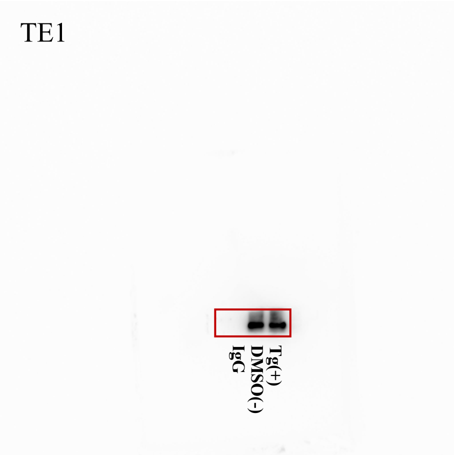


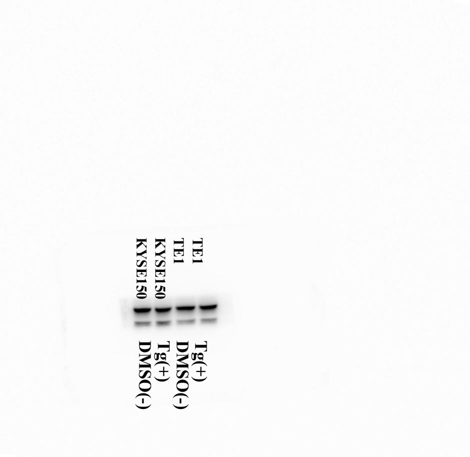


APMAP (protein bands of APMAP in KYSE150 and TE1 cells in Fig. 7H).

**T**

KYSE150

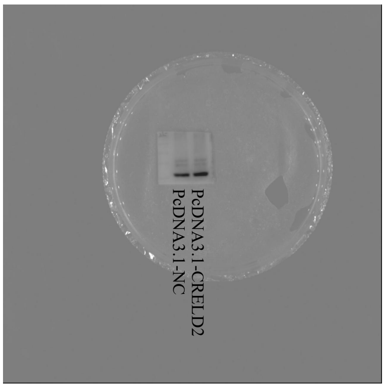


TE1

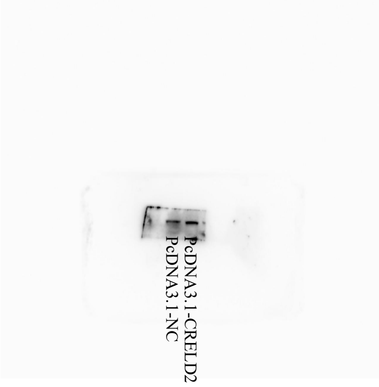


FN1 (protein bands of FN1 in CRELD2-overexpressing KYSE150 and TE1 cells in Fig. S4A).

KYSE150

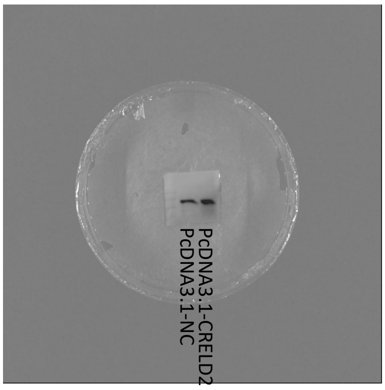


TE1

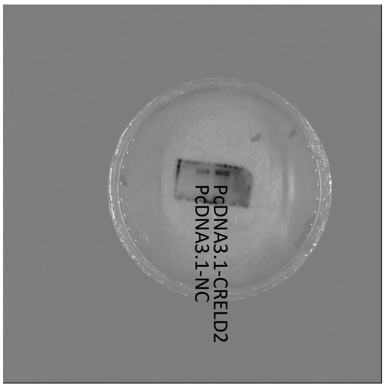


N-cadherin (protein bands of N-cadherin in CRELD2-overexpressing KYSE150 and TE1 cells in Fig. S4A).

KYSE150

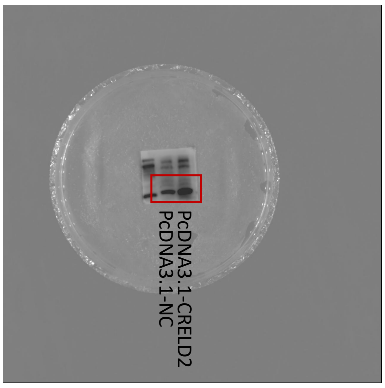


TE1

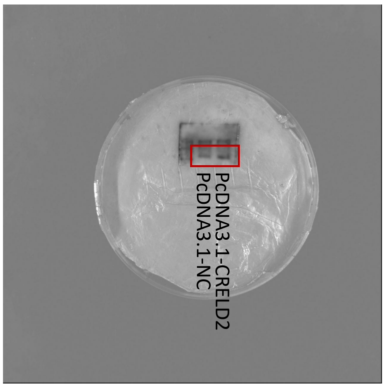


ZEB2 (protein bands of ZEB2 in CRELD2-overexpressing KYSE150 and TE1 cells in Fig. S4A).

KYSE150 TE1

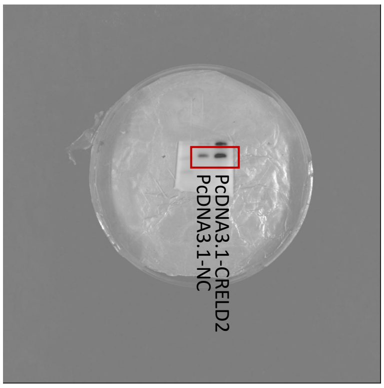


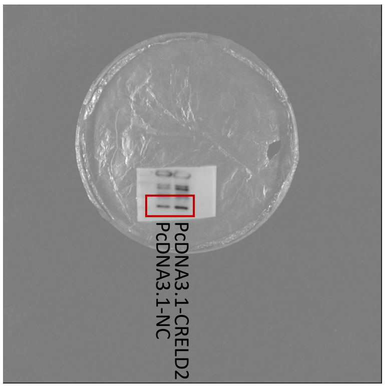


CCND1 (protein bands of CCND1 in CRELD2-overexpressing KYSE150 and TE1 cells in Fig. S4A).

KYSE150 TE1

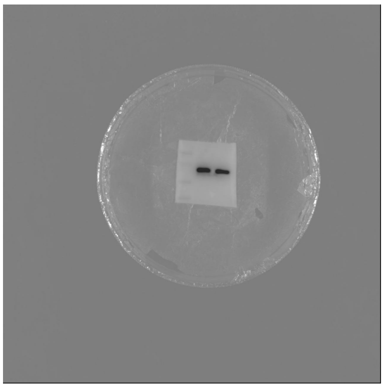

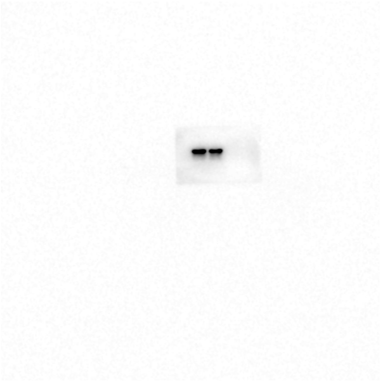

β-actin (protein bands of β-actin in CRELD2-overexpressing KYSE150 and TE1 cells in Fig. S4A).

**U**

KYSE150 TE1

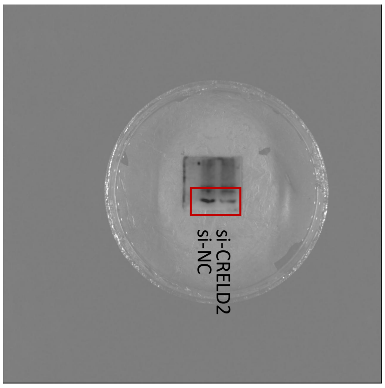

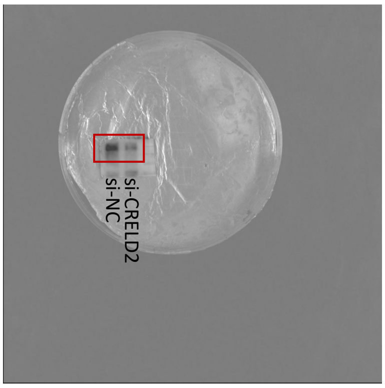


FN1 (protein bands of FN1 in CRELD2-knockdown KYSE150 and TE1 cells in Fig. S4B).

KYSE150 TE1

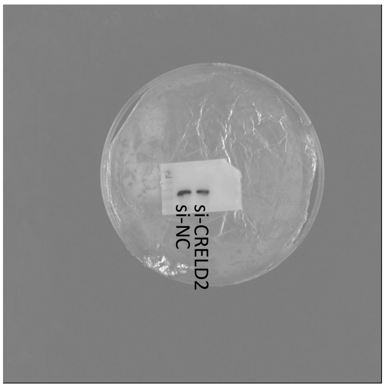

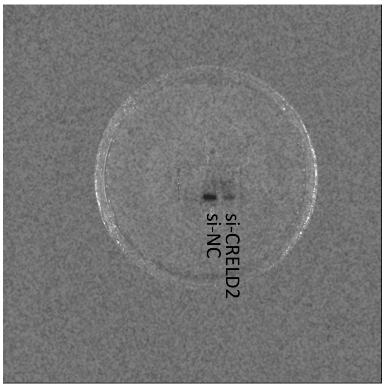


N-cadherin (protein bands of N-cadherin in CRELD2-knockdown KYSE150 and TE1 cells in Fig. S4B).

KYSE150 TE1

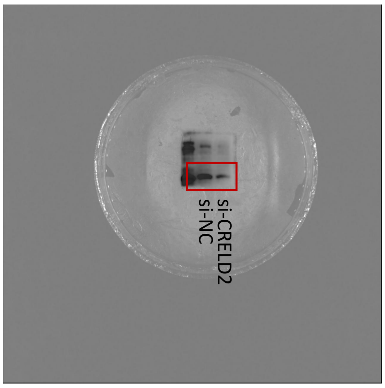

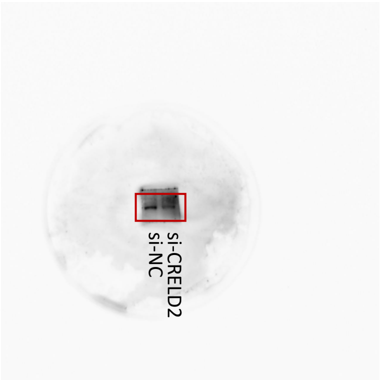


ZEB2 (protein bands of ZEB2 in CRELD2-knockdown KYSE150 and TE1 cells in Fig. S4B).

KYSE150

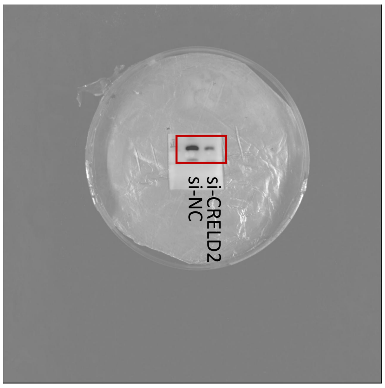


TE1

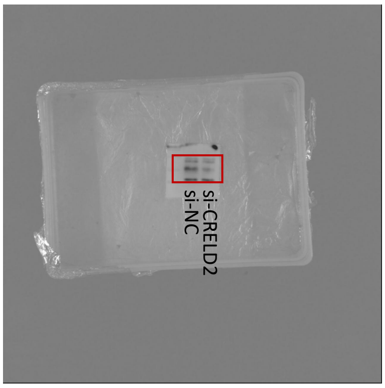


CCND1 (protein bands of CCND1 in CRELD2-knockdown KYSE150 and TE1 cells in Fig. S4B).

KYSE150

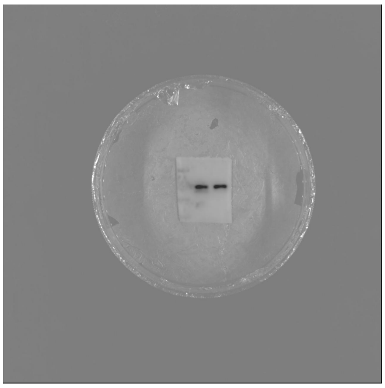

TE1

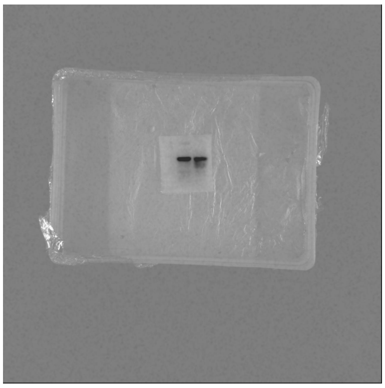

β-actin (protein bands of β-actin in CRELD2-knockdown KYSE150 and TE1 cells in Fig. S4B).

**V**

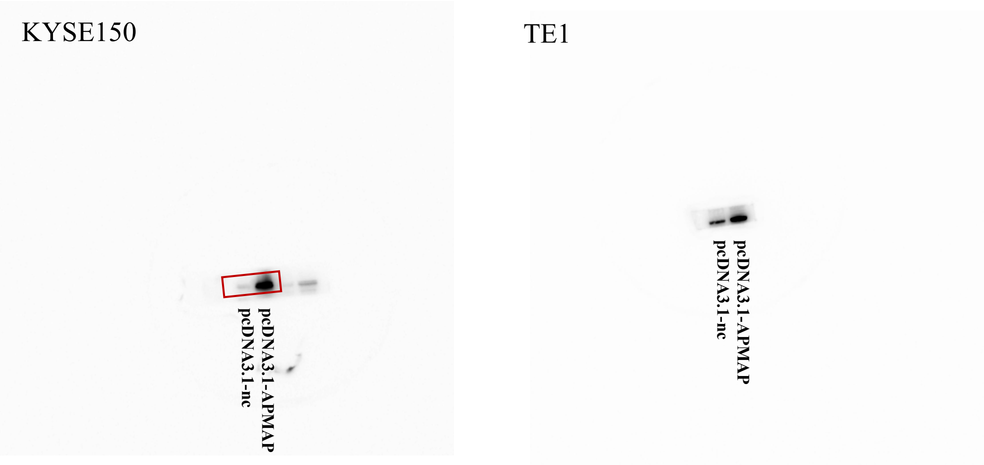


APMAP (protein bands of APMAP in KYSE150 and TE1 cells in Fig. S5B).

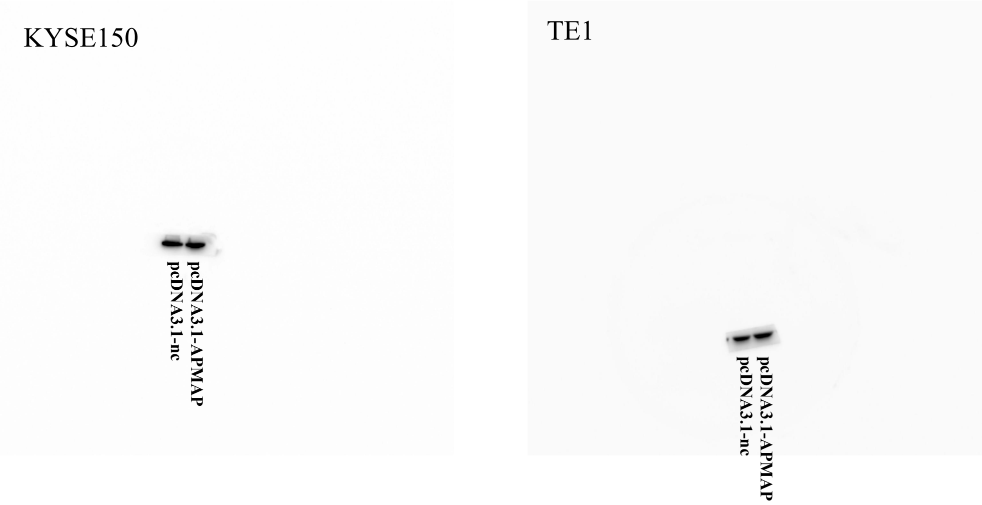


β-actin (protein bands of β-actin in KYSE150 and TE1 cells in Fig. S5B).

**The original images of western blots.** (A) The original images of the western blots in Fig. 1A. (B) The original images of the western blots in Fig. 1B. (C) The original images of the western blots in Fig. 1E. (D) The original images of the western blots in Fig. 1F and Fig. 1G. (E) The original images of the western blots in Fig. 2C. (F) The original images of the western blots in Fig. 2D. (G) The original images of the western blots in Fig. 2F. (H) The original images of the western blots in Fig. 2H. (I)

The original images of the western blots in Fig. 5A. (J) The original images of the western blots in Fig. 5D. (K) The original images of the western blots in Fig. 5E. (L) The original images of the western blots in Fig. 5H. (M) The original images of the western blots in Fig. 7B. (N) The original images of the western blots in Fig. 7C. (O) The original images of the western blots in Fig. 7D. (P) The original images of the western blots in Fig. 7E. (Q) The original images of the western blots in Fig. 7F. (R) The original images of the western blots in Fig. 7G. (S) The original images of the western blots in Fig. 7H. (T) The original images of the western blots in Fig. S4A. (U) The original images of the western blots in Fig. S4B. (V) The original images of the western blots in Fig. S5B.
